# Adverse Drug Reactions in Relation to Clozapine Plasma Levels: A Systematic Review

**DOI:** 10.3390/ph15070817

**Published:** 2022-07-01

**Authors:** Maria Skokou, Eleni A. Karavia, Zoi Drakou, Vassiliki Konstantinopoulou, Christina-Anna Kavakioti, Philippos Gourzis, Kyriakos E. Kypreos, Ourania Andreopoulou

**Affiliations:** 1 Department of Psychiatry, General University Hospital of Patras, School of Medicine, University of Patras, 26504 Patras, Greece; mskokou@upatras.gr (M.S.); zoidrakou@gmail.com (Z.D.); christiana-kav@hotmail.com (C.-A.K.); pgourzis@upatras.gr (P.G.); 2 Department of Pharmacology, School of Medicine, University of Patras, 26504 Patras, Greece; karaviae@hotmail.com (E.A.K.); venia45@yahoo.gr (V.K.); kkypreos@upatras.gr (K.E.K.); 3 Department of Life Sciences, School of Sciences, European University of Cyprus, Nicosia 2404, Cyprus

**Keywords:** clozapine, side effects, plasma levels, clinical pharmacology, Jadad scoring system, treatment-resistant schizophrenia, antipsychotics, neurological adverse effects, cardiological adverse effects, granulocytopenia

## Abstract

Clozapine is the gold standard for treatment-resistant schizophrenia. Serious and even life-threatening adverse effects, mostly granulocytopenia, myocarditis, and constipation, are of great clinical concern and constitute a barrier to prescribing clozapine, thus depriving many eligible patients of a lifesaving treatment option. Interestingly, clozapine presents variable pharmacokinetics affected by numerous parameters, leading to significant inter- and intra-individual variation. Therefore, therapeutic drug monitoring of plasma clozapine levels confers a significant benefit in everyday clinical practice by increasing the confidence of the prescribing doctor to the drug and the adherence of the patient to the treatment, mainly by ensuring effective treatment and limited dose-related side effects. In the present systematic review, we aimed at identifying how a full range of adverse effects relates to plasma clozapine levels, using the Jadad grading system for assessing the quality of the available clinical evidence. Our findings indicate that EEG slowing, obsessive-compulsive symptoms, heart rate variability, hyperinsulinemia, metabolic syndrome, and constipation correlate to plasma clozapine levels, whereas QTc, myocarditis, sudden death, leucopenia, neutropenia, sialorrhea, are rather unrelated. Rapid dose escalation at the initiation of treatment might contribute to the emergence of myocarditis, or leucopenia. Strategies for managing adverse effects are different in these conditions and are discussed accordingly.

## 1. Introduction

Efficacy and safety represent core aspects of medicinal treatments, and Hippocrates was probably the first to employ these concepts, in his aphorism “first, do no harm”, as also quoted in the Hippocratic Oath in the 5th century B.C.: “And I will use regimens to the benefit of the ill in accordance with my ability and my judgment, but from what is to their harm or injustice I will keep them” [1]. Some centuries later, Paracelsus stressed the significance of the drug dose for the emergence of adverse outcomes, saying, “What is there that is not poison? All things are poison and nothing is without poison. Solely the dose determines that a thing is not a poison” [2]. For modern psychiatrists, the case of clozapine encompasses the quest for optimal efficacy and safety in a dramatic mode, since this drug combines superior antipsychotic efficacy with serious and sometimes life-threatening adverse effects. Clozapine use has significantly improved the quality of life of treated patients and a strong association between clozapine use and reduced risk of mortality from natural causes [3], and all-cause mortality [4] has been reported, in those with treatment-resistant schizophrenia (TRS). For many patients, it has proved to be a lifesaving medicine. 

Clozapine (CLOZ) is the first of a class of antipsychotic compounds collectively termed as “atypical”, i.e., risperidone, olanzapine, quetiapine, aripiprazole, among others, all of which (with some exceptions) share D2 and 5-HT_2A_ blockage properties and are associated with fewer extrapyramidal side effects (EPSE) [5,6]. In fact, clozapine has a minimal incidence of EPSE, which was at first surprising, since researchers in the past related antipsychotic efficacy with the emergence of extrapyramidal symptoms [7]. It is proven superior to all other antipsychotics for the treatment of multiepisode schizophrenia and represents a first-line and gold standard option for TRS [8,9]. TRS is defined by consensus criteria as having positive and negative symptoms of at least moderate severity and at least moderate functioning impairment, with failure to respond to at least two different antipsychotic medications administered for at least 6 weeks at a minimum dosage equivalent to 600 mg chlorpromazine; this situation is encountered in about 30% of schizophrenia cases [10,11]. Clozapine is also indicated for recurrent suicidality in patients with schizophrenia or schizoaffective disorder and psychosis in Parkinson’s disease, irresponsive to other antipsychotics. Off-label uses include tardive dyskinesia and dystonia, treatment-resistant bipolar disorder, and schizophrenia with severe hostility [12]. According to the anatomical therapeutic chemical classification system (ATC) clozapine belongs to the N05AH02 class of drugs with a daily defined dose (DDD) of 300 milligrams per os [13].

Despite extensive preclinical research on its pharmacodynamics, its mechanism of action remains elusive. It is a tricyclic dibenzodiazepine, known to have weak dopamine-receptor-blocking activity at D1, D2, D3, and D5 receptors, but exerts highly potent D4 receptor antagonism [14]. Blockade of D4 is considered important for clozapine’s antipsychotic action, because of its abundance in the hippocampus and mesolimbic system [15]. Additionally, clozapine displays potent anti-adrenergic (α1 and α2), anticholinergic (Μ1, Μ2, Μ3, Μ5), antihistaminic (H1), antiserotoninergic (potent 5-HT_2A_ and 5-HT_2C_ receptor antagonist) and anti-GABAergic properties (GABA_A_) [14]. It also exerts partial agonism on M4 receptors and possibly modulates glutamatergic and GABA_B_ neurotransmission [14,16]. Remarkably, there is currently insufficient explanation for clozapine’s superior efficacy. Apart from its unique binding profile to various receptors, presumably over 35 different receptors, there has been demonstrated a positive effect on neuroprotection, through modulation of microglia activation [17]. On the other hand, the drug’s affinity for a variety of receptors accounts for several of its adverse effects, at least those that are considered dose-related. Muscarinic antagonism produces anticholinergic actions, in turn leading to tachycardia, constipation, urinary retention, and enuresis. Antagonism of α1 norepinephrine receptors may produce hypotension, sedation, bradycardia, and syncope. Antihistaminic action is associated with sedation and weight gain [18].

In terms of its pharmacokinetics, clozapine is almost thoroughly absorbed by the gut and undergoes extensive hepatic metabolism [19], mainly by CYP1A2 and to a lesser extent by CYP3A5 and CYP3A43 [20,21,22]. In vitro studies indicated that CYP2D6 and CYP2C19 have a minor role in clozapine metabolism [19,23,24]. Its most studied metabolites are norclozapine, (N-desmethyl-clozapine), considered active but to a lower extent than the mother molecule, and N-oxide -clozapine, which is inactive, but can convert back to clozapine [19]. Norclozapine (NCLOZ) has potent M_4_ cholinergic actions and is thought to possibly enhance cognition [25].

Clozapine demonstrates highly variable pharmacokinetics and for a given dose, plasma clozapine levels present large inter-and intra-individual differences affected by age, sex, genetics, dietary, clinical, pharmacological, drug interactions, and other parameters [26,27]. Therefore, prediction of plasma levels according to dose is impossible. In general, the dose regimen should be personalized, with the desirable plasma concentrations set for most patients at 350 ng/mL for optimal efficacy and safety [28].

Significant toxicity is generally associated with higher clozapine plasma levels. For example, existing evidence indicates that the incidence of seizures increases significantly at doses above 600 mg/day. Importantly, plasma levels over 1000 ng/mL have been linked to adverse central nervous system (CNS) effects and are not recommended due to a perceived risk of developing full-blown toxicity [29]. In the United Kingdom’s National Health Service (UK’s NHS) specific plasma monitoring protocols have been developed to this effect (i.e., https://www.hpft.nhs.uk/media/4116/clozapine-plasma-level-monitoring-guide-with-logos-mar19.pdf (accessed on 20 June 2022)) and dedicated clozapine clinical protocols have been established (i.e., http://www.rdash.nhs.uk/wp-content/uploads/2014/04/Clozapine-Clinic-Protocol-v3.pdf (accessed on 20 June 2022)). Recent reviews have investigated adverse effects in relation to clozapine plasma levels [30,31,32], such as peripheral adverse effects, neutropenia, EEG changes, and epileptic seizures, helping the search for a best-informed approach to clozapine use [33].

In the present systematic review, we discuss a full range of adverse effects of clozapine, in relation to plasma drug levels, classified by organ system. Our aim is to provide comprehensive and concise guidance to clinicians, researchers, and clinical pharmacologists on the safe use of clozapine, helping to overcome barriers responsible for its underutilization.

## 2. Results

### 2.1. General Considerations during Literature Search and Evaluation

**Figure 1:**
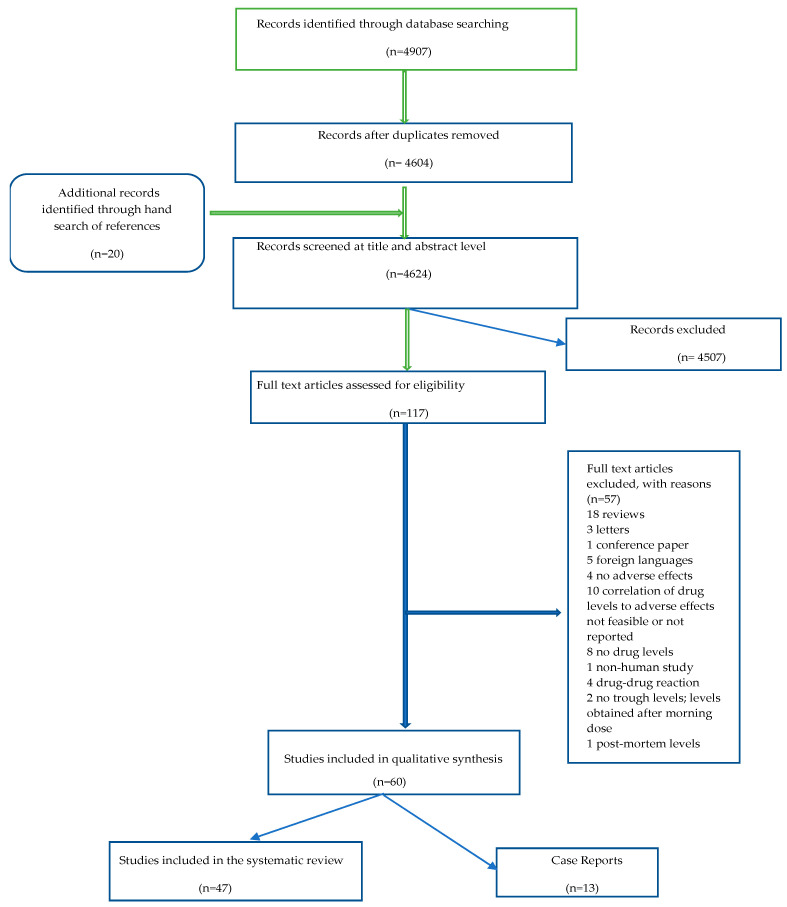
Flowchart of search strategy and article selection.

The search strategy followed by the exclusion process yielded a total of 60 articles out of which 47 were original research papers and 13 were case reports, as shown in the flowchart of Figure 1. Although case reports were not included in the review, they are listed in Supplementary Table S1A, because rare adverse effects are not likely to be reported in the usually employed sample sizes. For most of the studies, clinical evidence was classified as low quality, due to numerous limitations. In most studies, blood samples were drawn immediately prior to the morning dose of clozapine, or 8 to 16 h after the night-time dose. Eleven studies did not mention explicitly the time of sampling. Many studies were performed in a small or small to medium size sample, ranging from 6 to 190 patients receiving clozapine. Correlation of clozapine or metabolite levels with dose was calculated in thirteen studies [34,35,36,37,38,39,40,41,42,43,44,45,46]; nine reported a positive correlation between dose and plasma clozapine levels alone or with metabolite levels as well [34,35,36,37,39,41,42,43,44] and two reported no correlation with clozapine and norclozapine levels [45,46]. Sporn et al. [40] found a positive correlation of clozapine dose with norclozapine but not clozapine plasma levels, and Mauri et al. [38] reported that clozapine dosage correlated with clozapine but not norclozapine plasma levels. Furthermore, 27 studies examined the correlation of clozapine dose with adverse effects; of them, 19 found no correlation between dose and side effects studied [22,35,36,37,38,41,47,48,49,50,51,52,53,54,55,56,57,58,59,60,61]. The rest [34,43,62,63,64,65,66,67] reported positive correlations with side effects, albeit weaker or to a lesser extent than correlations with plasma clozapine levels. The time point in the patients’ treatment with clozapine was variable (initiation of clozapine, maintenance treatment, years after clozapine initiation), and the mode of titration in patients starting therapy ranged from rapid to slow. Differences existed in terms of comedications, which varied from none, in some prospective studies with wash out periods, up to different classes of psychotropics (mood stabilizers, antipsychotics, antidepressants, benzodiazepines). Many, but not all studies, used scales for detecting side effects and for measuring their severity, instead of rating adverse effects as present vs. absent. These data are presented in Supplementary Table S1B, except for comedications, which are reported in the results sections below.

### 2.2. Method of Plasma Clozapine Measurement

Eleven of the selected research papers do not specify how plasma clozapine concentrations were quantified. In the remaining, which describe a method of clozapine measurement, high-performance liquid chromatography (HPLC) and reversed-phase HPLC (RP-HPLC) combined with ultraviolet (UV) detection were reported as the most common methods. Gas chromatography (GC) was also used. Liquid chromatography (LC), LC coupled with mass spectrometry (LC-MS), LC coupled with tandem mass spectrometry (LC-MS/MS), and gas chromatography-mass spectrometry (GC-MS) were utilized in fewer studies (Table 1).

### 2.3. Overall or Combined Adverse Effects

Four studies examined associations between clozapine levels and a total number of side effects or combinations of side effects (Table 2, Table S2). Frazier et al. [43] found an association between norclozapine (r = 0.6, *p* = 0.002), and norclozapine + clozapine levels (r = 0.4, *p* = 0.002) and the total number of moderate and severe side effects, in a small (*n* = 6) pediatric sample, at 6 weeks of therapy. Yusufi et al. [56], by applying the Antipsychotic Non-Neurological Side Effects Rating Scale (ANNSERS) to a sample of adult patients at maintenance therapy with clozapine, found that the drug levels correlated weakly with the number of moderate and severe side effects (r = 0.23, *p* < 0.03) and the total scale score (r = 0.29, *p* < 0.004). More than three-quarters (77%) of the patients had at least one and 28% had at least three side effects that were rated as moderate or severe. Further, Spina et al. [46], showed a small but not significant increase in the incidence of side effects, in patients with higher clozapine levels (440 ± 125 ng/mL) at 12 weeks of treatment. Another study employing a different scale, the Systematic Assessment for Treatment Emergent Effects (SAFTEE), reported no correlation between total scale score and drug levels [41].

### 2.4. Nervous System Adverse Effects

#### 2.4.1. Εlectroencephalographic (EEG) Abnormalities and Seizures

Seven studies addressed the issue of electroencephalographic (EEG) changes and/or seizures in relation to clozapine levels. Of these, two [35,47] examined patients at the maintenance phase, a few years after therapy initiation, while all others refer to patients at the early treatment phase (a maximum of 12 weeks). In a prospective, randomized clinical trial (Jadad score = 2) [34], 50 patients with treatment-resistant schizophrenia or schizoaffective disorder, were randomly assigned into three groups according to their clozapine plasma levels: group I: 50–150 ng/mL, group II: 200–300 ng/mL, group III: 350–450 ng/mL. Three patients (6%) exhibited seizures, with two having a prior history of seizures (assigned to group II) and one to group III. After excluding five patients who were on valproate, in the rest of the patients (*n* = 45) slowing was reported in 53% of them and was significantly more frequent in group III (82%), compared to groups I (20%) and II (26%). Spike/sharp activity (13%) and sleepiness (31%) were not significantly different among groups. EEG changes were not significantly different between responders and non-responders. Sleepiness correlated significantly with slowing, but not with clozapine serum levels, possibly due to a lower sensitivity of nurse observation, according to the authors.

Haring et al. [58] studied the occurrence of EEG changes in a group of 29 hospitalized patients treated with clozapine, in the context of a prospective clozapine drug monitoring program. Patients were classified into two subgroups based on EEG findings: in the first (53% of patients) pathological changes on the EEG were noted, while in the second (47% of patients), minimal or no changes were observed. This difference correlated with marked differences in plasma clozapine levels between groups (81.6 ± 64.6 ng/mL vs. 235.7 ± 169.8 ng/mL for patients with minimal versus pathological changes in EEG, respectively, *p* = 0.0009). No seizures or severe alterations were observed during the study. Administered dose, duration of treatment, sex, age, and weight had no influence on the induction of pathological EEG patterns. Similar results were found in a more recent retrospective study, where patients with clozapine levels over 600 ng/mL had a higher rate of EEG abnormalities than those below 600 ng/mL (93.8% vs. 65.5%, *p* = 0.02); again, dose, duration of illness or treatment, age, norclozapine levels did not correlate with EEG abnormalities [47]. In another study [35] (*n* = 30, chronic schizophrenia) with higher plasma clozapine levels (median: 1076 ng/mL, range: 706 ng/mL–1882 ng/mL), the severity of EEG changes was positively correlated with plasma clozapine but not norclozapine levels. Eighty-three percent of the subjects presented EEG abnormalities, similarly to the higher serum level group (group III) of the study by Freudenreich et al. [34].

In a Chinese study [42] (*n* = 51), similar incidence of EEG abnormalities (81.58%) was found on week 6 of observation but the severity was not assessed, and no correlation was found between incidence and clozapine levels (range: 100–1220 ng/mL, mean: 470.20 ± 234.23 ng/mL). Centorrino et al. [41] (*n* = 44, chronic patients) reported the absence of seizures in their sample but no EEG recordings were conducted (median clozapine levels: 291 ng/mL, range: 15–726 ng/mL). Finally, in a pediatric population [40] (*n* = 54) with childhood-onset schizophrenia (COS) reporting side effects at week 6 of observation, seizures were found in 6%, EEG abnormalities and slowing in 11%, and epileptiform discharges in 11% of study participants. Again, severity was not assessed and correlation with clozapine levels was not found (range of clozapine levels: 455 ± 285.1 ng/mL, and during follow-up: 395.7 ± 206.0 ng/mL).

#### 2.4.2. Cognition

Rajji et al. [59] found that high cognitive impairment as measured by an assessment tool for global cognition was more frequently encountered in patients with higher age, higher clozapine levels (534.7 ± 237.7 ng/mL), and a higher clozapine/norclozapine ratio. Causality could not be established due to several limitations in the study design, but the finding could be supported by the partial agonism exerted by norclozapine on muscarinic receptors, contrary to the antagonistic muscarinic properties of clozapine. In another study, however [68], no significant correlation between plasma clozapine levels and cognitive performance could be established, except for a trend, on executive tests. Similarly, Yusufi et al. [56] found memory problems and lack of concentration in 38% of patients, but no correlation with plasma clozapine levels could be established. The Jadad score for all three studies suggested a low quality of clinical evidence.

#### 2.4.3. Extrapyramidal Side Effects

VanderZwaag et al. [39] reported improvement of bradykinesia/rigidity, tremor, and akathisia, during a 12-week treatment period, irrespective of plasma levels; as for dyskinesia, 80% of patients who had scores >1 on the Abnormal Involuntary Movement Scale (AIMS) showed improvement over time. Liu et al. [82] reported mild rigidity in 8.1% and tremor in 3.2% of patients but as these might have been due to delayed effects of the previous therapy with haloperidol they were not reported as side effects of clozapine (mean levels: 598 ± 314, range 111–1585 ng/mL).

Yusufi et al. [56] found parkinsonism, akathisia, and tardive dyskinesia in 18%, 4%, and 5%, respectively, with no significant correlation with plasma levels (530 ± 370 ng/mL). In a pediatric sample [40] akathisia was reported on week 6 (15%), with no correlation with levels (455 ± 285.1 ng/mL).

A cohort study (*n* = 37) [83] was conducted for a period of 6 weeks and the mean doses of clozapine differed each week. Similarly, plasma clozapine levels were also different (week 1:450 ng/mL, week 2:650 ng/mL, week 3:450 ng/mL, week 4:575 ng/mL, week 5:575 ng/mL, week 6:663 ng/mL). The study indicates that none of the 7 patients without tardive dyskinesia (TD) developed TD in an average of ~6.9 months and there was no significant increase observed in the Hillside Modified version of the Simpson Dyskinesia Scale (SDS) for the non-TD patients. Nervous system adverse effects in relation to CLOZ levels are shown in Table 3 and Supplemental Table S3.

### 2.5. Psychiatric Adverse Effects

Obsessive-compulsive symptoms (OCS) in relation to plasma clozapine levels were examined in one retrospective study conducted in Taiwan [60] (*n* = 102). Thirty-nine patients (38.2%) manifested OCS. In 29 patients (28.4%) OCS was classified as clozapine-induced, with an average latency period of 39.8 ± 22.5 months of time-to-event. Plasma clozapine levels were remarkably higher in patients with OCS than in those without (595.1 ± 364.9 vs. 433.5 ± 252.8 ng/mL, *p* = 0.001) though no significant difference existed in the daily administered clozapine dose. By a quartile odds ratio comparison, patients with the highest plasma clozapine levels presented a four-fold higher probability of developing OCS.

Moreover, a cross-sectional study of 237 patients where serum clozapine and norclozapine plasma levels were available for 190 subjects, showed that the depression/anxiety factor score (comprising items such as difficulty in concentrating, tension, difficulty remembering things, depression, restlessness, difficulty getting to sleep) was significantly higher in patients with combined clozapine and norclozapine serum concentrations of at least 719 ng/mL (*n* = 92) compared to patients with lower concentration (*n* = 95). The correlation was not significant for sedation, dreaming, lack of emotion, and sympatichotonia factor scores [75]. In another study, night-time sleep problems were found in 32% of patients [56]. Mean clozapine levels were 530 ± 370 ng/mL and no correlation with levels was found [56].

In summary, a positive correlation between clozapine plasma levels and OCS has been reported; a correlation with depression/anxiety constellation of symptoms was noted, but the causality remains unclear (Table 3).

### 2.6. Cardiovascular System

#### 2.6.1. QTc Prolongation

No significant correlation was found in the study of Wong et al. [42], where mean clozapine levels were 470.20 ± 234.23 ng/mL, but a caveat of the work was that baseline measurement was conducted during the tailing off of previous antipsychotic medications that could have an impact on QTc as well. Similarly, Grande et al. [79] found no significant correlation between clozapine levels > 400 ng/mL and QTc prolongation, in a retrospective study of 82 patients. The best predictors of QTc prolongation were QTc before treatment, age, and heart rate > 95 beats/min.

#### 2.6.2. Sudden Death, Myocardial Function, Myocarditis, and Pericarditis

More recently, results of a longitudinal follow-up cohort study in Australia (*n* = 503) were reported [80]. The endpoints of the study were discontinuation of clozapine, all-cause mortality, sudden death, the incidence of clinical myocarditis, and time to myocarditis. Myocarditis was diagnosed by troponin I elevation and evidence of left ventricular systolic dysfunction. After clozapine initiation, incidence of sudden death was 2% (*n* = 10, time-to-event: 5 ± 4 years, and of myocarditis 3% (*n* = 14, time-to-event: 15 ± 7 days). Average plasma clozapine levels for the entire cohort were 475 ± 236 ng/mL compared to 439 ± 198 ng/mL in subjects who suffered a sudden death and 297 ± 152 ng/mL in subjects with myocarditis. Based on these, myocarditis or sudden death cannot directly be linked to clozapine blood levels. Nevertheless, plasma levels found in patients with myocarditis, in relation to the duration of clozapine exposure, point to a rapid mode of dose escalation. On the other hand, sudden death was correlated with recent weight gain and morbid obesity (70% of the patients gained weight) but not with prolonged QTc which was found normal, although ventricular arrhythmias were documented in half of those patients (*n* = 5).

Echocardiographic measures have been performed in a cohort of 15 patients with schizophrenia, receiving a daily clozapine dose titrated to 100 mg. Titration took place over a period of 4 weeks, starting at 25 mg per day at week 1 and increasing dose weekly by 25 mg increments [76]. The authors reported less efficient left ventricular function, without overt clinical manifestations in 60–80% of the sample, at rather low clozapine and norclozapine plasma levels (124 ± 70.8 ng/mL and 52.3 ± 35.7 ng/mL, respectively). No correlation between echocardiographic findings and drug or metabolite levels could be established.

#### 2.6.3. Pulse and Blood Pressure Irregularities

Oyewumi et al. [64] reported a trend toward autonomic dysregulation during titration of clozapine in the first 8 weeks of therapy (*n* = 37). Small changes in temperature, pulse rate, and blood pressure, could relate to increased cardiovascular risk. Average plasma clozapine and norclozapine concentration and the norclozapine/clozapine ratio were 63.5 ± 46.0 ng/mL, 38.7 ± 30.8 ng/mL, and 0.65 ± 0.31 ng/mL, respectively, at week 1, and 379.5 ± 156.5 ng/mL, 249.6 ± 94.7 ng/mL and 0.70 ± 0.16 ng/mL, respectively, at week 8. The norclozapine/clozapine ratio was a significant covariate of the erect and supine systolic blood pressure (BP) (*p* < 0.001) and the standing diastolic BP (*p* < 0.002). A higher dose of clozapine and higher norclozapine levels were correlated with lower temperatures, (mean temperature was 36.6 ± 0.9 °C and 35.9 ± 0.6 °C at weeks 2 and 7, respectively) and higher norclozapine/clozapine ratio was significantly but weakly related to higher BP measures. Another study [44] on a very small subset of patients (*n* = 5) with known clozapine levels between 200–300 ng/mL found a positive relationship with severity of orthostatic dysregulation at day 6 (*p* < 0.05) and day 10 (*p* < 0.05) of treatment. The mean severity score was higher on day 3, whereas an adaptation occurred after 10–20 days of treatment. In the rest of the studies [35,39,56,82], orthostatic hypotension was found in 5–27% of patients, with no significant correlation with plasma clozapine concentration.

Many studies reported tachycardia (>100 bpm) in 23–51% of patients [35,39,41,56,82]. In one of these [35], in adult patients with chronic schizophrenia (*n* = 30), increased pulse rate (>80 bpm) was also reported in 73% of the participants in contrast to Spina et al. [46], who reported a much lower rate of 4.4%. Nevertheless, no association with drug levels was found. A more recent study employing 24 h ambulatory ECG recording (Holter) in 30 patients with HR >100 bpm did not find any relationship between clozapine levels and HR variables; HR was consistently very high during day- and night-time. Data concerning patients without baseline tachycardia were not included [48].

Heart rate variability (HRV) correlated negatively with cardiovascular risk. Clozapine levels appeared to inversely correlate with HRV parameters, and this influence was stronger than the influence of age [72]. This finding was later replicated by Eschweiler et al. [73], who reported that all patients with clozapine levels below 350 ng/mL had CV > 3.2%, contrasting patients with higher levels who all had CV < 3.2% (*p* < 0.001), setting this level as a separating point.

In pediatric populations, tachycardia was reported in 28–67% of patients [40,43] with no apparent correlation to plasma clozapine levels. Furthermore, Sporn et al. [40] reported hypertension (>140/90 mmhg) at a rate of 6% and orthostatic hypotension at a rate of 7%, which again did not present any correlation with plasma drug levels, 6 weeks after treatment initiation. In this study orthostatic hypotension was defined as a fall in systolic/diastolic BP >20/10 mmhg or an increase in heart rate >30 bpm within 3 min of obtaining the upright position.

In summary, QTc prolongation appears independent of plasma clozapine levels. Myocarditis, usually an idiosyncratic reaction, could relate to higher plasma clozapine levels, or rapid escalation of these levels, during initiation of therapy. Sudden death better correlates with other factors rather than plasma clozapine concentration. Importantly, HRV is negatively correlated to plasma clozapine levels. Finally, orthostatic dysregulation or hypotension and temperature dysregulation, but not tachycardia, show a positive correlation with clozapine plasma levels. Studies reporting cardiovascular adverse effects are presented in a tabular form in Table 4 and complementary Table S4.

### 2.7. Metabolic Adverse Effects

Eleven studies reported metabolic side effects in relation to plasma clozapine levels. Specifically, these studies assessed weight gain, metabolic syndrome, glucose, and lipid abnormalities.

#### 2.7.1. Weight Gain

Three studies evaluated weight gain in relation to clozapine levels and found no significant association [50,63,71]. The sample size in all three studies was small to moderate (*n* = 61–74) and the duration of clozapine exposure varied from 12 weeks [71] to 16–48 weeks [63] to 6 months [50]. Similar results were obtained in another small pediatric sample [43].

However, a significant positive association between norclozapine levels and weight gain was reported by Lu and coworkers [71] who studied fluvoxamine add-on therapy as a way of reducing this adverse effect. The rationale of this approach is based on the observation that fluvoxamine lowers norclozapine levels. Similarly, norclozapine levels were significantly and positively associated with weight gain in nonsmokers (*n* = 8) in the study of De Leon et al. [63] after controlling for baseline BMI, sex, and clozapine levels.

Two studies reported the existence of excess body weight or BMI, as common physical features among their subjects and considered them an indirect indication of weight gain [41,51]. However, no direct evaluation of weight gain could be performed since baseline weight was not available. Vasudev et al. [51] found that clozapine levels correlated with metabolic syndrome. The authors calculated an 11% odds ratio increase per 100 ng/mL of clozapine blood level. On the other hand, Anderson et al. [74] reported a positive correlation between clozapine levels and BMI, in a retrospective study investigating sex differences in clozapine levels and metabolic parameters as a primary focus. Females had significantly higher BMI and clozapine levels compared to men.

#### 2.7.2. Dyslipidemia

According to the study by Subramaniam et al. [65], clozapine levels could not predict lipid levels, total cholesterol (TC), or triglyceride (TRG) levels. Along the same line, a prospective observational study that examined the associations between therapeutic response to clozapine and lipid dysregulation in 49 patients [81], showed that increases in TRG levels were associated with clinical response to clozapine treatment (*p* < 0.001), but plasma clozapine concentrations did not correlate with TRG increase. Similarly, no correlation between TRG and clozapine levels was found in two other studies [71,77]

On the contrary, TRG was found to correlate to plasma clozapine concentration (*p* = 0.03) in another, cross-sectional study [70]. Hyperlipidemia was found in 40–60%, but again no correlation was found between cholesterol, high-density lipoprotein cholesterol (HDL-C), or LDL cholesterol (LDL-C) and plasma clozapine levels. Anderson et al. [74] found no association between clozapine levels and HDL-C. Lu et al. [71] reported a positive correlation between serum TRG and norclozapine but not clozapine concentration. Lipid elevation was found to associate with clozapine levels and norclozapine/clozapine ratios by Melkersson et al. [45]. Further, TC, but not HDL-C or LDL-C, correlated positively with plasma clozapine levels in a case-control study [77].

#### 2.7.3. Hyperglycemia, Hyperinsulinemia, Insulin Resistance

Hyperglycemia has been found to positively correlate [74] or not related [65,70,71,77] to clozapine plasma levels. In the study of Subramaniam et al. [65], female gender (*p* = 0.02) and age (*p* = 0.04) but not clozapine concentrations were found to be significant predictors of glucose levels. Anderson et al. [74] have compared clozapine plasma levels and hyperglycemia between the two sexes and found that women had significantly higher clozapine levels and blood glucose than men, therefore indirectly pointing to a positive correlation between drug levels and levels of glucose. The findings of the two studies should be interpreted with caution because the groups compared differ in race [65] and sex [70]. Lu et al. [71] found that serum glucose was positively correlated with norclozapine, but not clozapine levels.

Insulin levels were positively correlated to clozapine serum concentration [49]. In the study by Melkersson and Dahl [70], hyperinsulinemia was present in 30–60% of patients and hyperglycemia in 10–30%. Plasma levels of insulin and C-peptide were positively correlated with plasma clozapine levels (*p* = 0.03 and *p* = 0.04, respectively). Insulin elevation and insulin resistance positive correlation with clozapine levels as well as norclozapine/clozapine ratios were also reported in a later study [45].

All studies, with the exception of one [63], were of low quality.

### 2.8. Endocrine System

Compared to typical antipsychotics, the effect of clozapine on prolactin (PRL) secretion is 5 to 10 times lower, according to a double-blind dose–response study. For every 100 ng/mL increase in plasma clozapine levels (clozapine plasma levels: 400–1600 ng/mL), average increments in prolactin levels of 0.45 ng/mL in females and 0.15 ng/mL in males were recorded. These effects were more evident in females, but no symptoms associated with hyperprolactinemia were recorded [66]. In another study, menstrual disturbances (period pains, reduced frequency of periods) were not associated with combined clozapine + norclozapine concentrations [75]. A negative correlation between clozapine levels and fT3 (r = −0.385, *p* = 0.021), but no correlation with TSH, fT4, or PRL, was more recently reported [77].

Studies relevant to metabolic and endocrine system side effects are shown in Table 5 and Supplemental Table S5.

### 2.9. Gastrointestinal System

Studies relevant to gastrointestinal adverse effects are shown in Table 6 and Supplemental Table S6.

#### 2.9.1. Hypersalivation, Nocturnal Sialorrhea, Drooling

Two studies examined hypersalivation in relation to clozapine levels and failed to establish a significant association. According to the first study, (cross-sectional, *n* = 44) nocturnal sialorrhea (observed in 80% of the subjects), drooling and parotid swelling were among the most common side effects [41], with no significant correlation between these and mean clozapine and norclozapine levels in serum. In the other study, (prospective, *n* = 45), hypersalivation was observed in just a small proportion of the cohort (*n* = 3) [46]. Mean clozapine levels in patients with hypersalivation were higher than those in patients without the side effect (440 ± 125 ng/mL versus 359 ± 205 ng/mL, *p* = 0.212), but the difference did not reach statistical significance (*p* = 0.199).

#### 2.9.2. Hepatotoxicity

Hummer et al. [52], in their prospective study, found a significantly positive correlation between plasma clozapine levels (165.4 ± 163.4 ng/mL) and increases in serum glutamic-pyruvic transaminase (SGPT). Increases in SGPT were more prominent during weeks 1–6, and thereafter generally decreased. Yet, three other, cross-sectional, studies [35,65,77], failed to find such an association. Incidence of increased γ-GT was 60% and the incidence of increased AST, ALT, and ALP was 15–25%, irrespective of duration of treatment, clozapine, norclozapine, or norclozapine/clozapine ratio levels [35]. Liver enzyme activity was also not correlated with clozapine serum concentration, in two studies on pediatric samples [40,43] with childhood-onset schizophrenia. In the latter (very small sample, *n* = 6), one patient demonstrated increased hepatic transaminase concentrations.

#### 2.9.3. Constipation

Centorrino et al. [41], did not find a statistically significant association between constipation and clozapine concentration. A very interesting, cross-sectional study found a strong association between clozapine use and gastrointestinal hypomotility, and, in fact, higher clozapine levels correlated with longer colonic transit times (CTT) [53]. On the other hand, in a study where laxative use served as an indirect marker for constipation [54], it was demonstrated that laxative users had 29% higher norclozapine concentrations compared with non-users. Clozapine levels did not differ significantly between the two groups.

### 2.10. Hematological System

Four studies examining leucocyte (WBC) and neutrophil (NEU) counts in relation to clozapine levels yielded negative results [35,36,37,69]. No case of agranulocytosis was reported in an open-label study with seven chronic paranoid schizophrenic patients [84]. Similarly no correlation was found with eosinophilia [37], increased erythrocyte folate concentration [35], lymphocyte number [37,77], hemoglobin (HGB) levels and hematocrit [37]. A weak positive correlation was noted between clozapine levels and platelet count (PLT) between week 2 and 4 after clozapine initiation and the clinical significance is unclear [37]; in contrast, Gharab et al. [77] found an inverse correlation between clozapine levels and PLT (r = −0.362, *p* = 0.025), HGB (r = −0.342, *p* = 0.025) and NEU (r = −0.385, *p* = 0.017), during chronic therapy. Red blood cell count (RBC) (*p* = 0.020), mean corpuscular hemoglobin (MCH) (*p* = 0.036), HGB (*p* = 0.015), NEU (*p* = 0.034), PLT (*p* = 0.005), mean platelet volume (MPV) (*p* = 0.003) were significantly lower in clozapine-treated patients compared to controls, whereas mean corpuscular volume (MCV) was significantly higher (*p* = 0.002). Mauri et al. found a positive correlation between clozapine levels and NEU (r = 0.26, *p* = 0.001). Still, WBC showed no correlation [38,77]. On the other hand, Vaquero-Baez et al. [67] reported a negative correlation between clozapine levels and WBC (r = −0.725, *p* = 0.001) and NEU (r = −0.631, *p* = 0.004).

A few studies examined the correlation of hematological disorders with norclozapine plasma levels. Of these, three reported no correlation to WBC or NEU counts [36,41,67]. Smith et al. [78] found a positive correlation between NEU count and norclozapine as well as norclozapine/clozapine ratio (*p* = 0.002, *p* = 0.04, respectively, in mixed-model analyses). Mauri et al. [38] showed that norclozapine was positively (r = 0.20, *p* = 0.01) and norclozapine/clozapine ratio negatively correlated (r = −0.26, *p* = 0.002) with NEU count. Turning to pediatric samples, neutropenia, observed in a significant proportion of the sample (6–16.5%) was not correlated to clozapine or norclozapine levels [40,43] (Table 7 and Table S7).

### 2.11. Genitourinary System

One study [62] that examined anticholinergic side effects in relation to clozapine and resultant antimuscarinic activity serum levels, found no correlation between enuresis with antimuscarinic activity. Other studies of lower quality also do not report any relevant association in adults [41] or in samples of children and adolescents with childhood-onset schizophrenia [40,43] (Table 7 and Supplemental Table S7). Sexual disturbances, namely diminished sexual desire and functional disturbances were influenced by clozapine levels, in males, but not in females; furthermore, such influence was not found for menstrual disturbances, dry vagina, gynecomastia, galactorrhea [57].

### 2.12. Other Adverse Effects

Clozapine-treated patients had higher anticardiolipin antibodies (aCL) (IgG and IgM isotypes) levels compared to healthy controls while there was a significant positive relationship between serum IgM aCL and serum clozapine level (r = 0.461, *p* = 0.001) [55] (Table 7). Further, no correlation was noted between B12, Na, K, urea, creatinine levels, and clozapine concentrations [77].

## 3. Discussion

Usually, clozapine ADRs are type A (augmented) reactions that result from an exaggeration of the drug’s normal pharmacological actions when given at the usual therapeutic dose and are normally dose-dependent. This may be due to elevated plasma levels of clozapine or its active metabolite norclozapine. Nevertheless, some adverse reactions are type B, meaning they are unpredictable, rare, and idiosyncratic reactions, largely independent of plasma drug levels. Table 8 summarizes adverse drug reactions and their relation to plasma clozapine levels.

The interaction of clozapine with various receptor systems in the central nervous system justifies not only its increased effectiveness but also the induction of type A ADRs related to its use. Indeed, its competitive effects on α-adrenergic receptors are linked to the frequent occurrence of nocturnal enuresis in patients, while, a competition specifically of α1 receptors is associated with the occurrence of orthostatic hypotension, sedation, and dizziness [85,86]. Blockade of the α1 receptor in combination with the anticholinergic properties of clozapine contributes to the occurrence of tachycardia [86,87]. Binding to adrenergic α2 and muscarinic M4 receptors contributes to salivation, while binding to muscarinic M3 may be related to insulin regulation and the development of hyperglycemia [86,88]. Similarly, binding to muscarinic M1 receptors is mainly associated with the occurrence of constipation, but also suppression and paralytic ileus [86]. Binding to H1 receptors is also associated with the occurrence of suppression, while blockade of both H1 and serotonin 1A, 2A, and 2C receptors contribute to increased appetite and weight gain [85]. Finally, blockade of D2 receptors may contribute to the development of malignant neuroleptic syndrome and, rarely, motor disorders [85,86].

The induction of clozapine type B ADRs remains a mystery. One such ADR is agranulocytosis which appears even at very low plasma levels of clozapine during the first few days or weeks of its use. In this case, the metabolism of clozapine to nitrenium ion by liver microsomes, peripheral blood neutrophils, and their bone marrow precursors is believed to play a crucial role. This toxic metabolite has been shown to covalently bind to neutrophil proteins and this interaction is believed to trigger toxicity, though the precise mechanism remains unclear [89]. Another potential reason for type B ADRs may be a local build-up of clozapine concentration within certain cells and tissues which may be idiosyncratic and may not correlate directly with plasma levels. This mechanism may explain leukocytopenia in some patients even at very low plasma levels of clozapine [90].

In practical terms, for type A ADRs, TDM can offer quite valuable guidance, aiding dose adjustments for optimal efficacy and safety. For some level-related adverse effects, such as severe constipation or EEG slowing, pointing to some degree of encephalopathy, levels should be lowered, by reducing the administered dose. For those type A ADRs where, although level-related, reducing dose is not possible or mandatory—for example, because efficacy is compromised—or for type B ADRs that are not related to levels, clinical strategies for their management must be a priority. Such situations are, for instance, adding on an SSRI for obsessive compulsive symptoms [91], or advising behavioral measures of the morning activity to counteract sedation [92]. Slow and careful titration is best in all cases, especially for neutropenia and myocarditis, which may involve immune mechanisms [33,93]. Needless to say, the art of the pharmacological treatment of each individual patient rests on the treating clinician, who uses TDM in the context of a unique therapeutic relationship.

Most of the studies in our work suffered significant limitations when evaluated by the Jadad grading system, preventing concrete conclusions. This fact partially mirrors the difficulty of conducting studies designed as randomized and double-blind, since serious ethical issues are raised with randomization and blindness [94]. On the other hand, real-world design of clinical trials can also offer useful insights into various clinical matters, including efficacy in real-world settings, add-on strategies, comorbidities, and drug safety [95].

During our search and clinical evidence evaluation, a considerable heterogeneity among studies was observed with regards to patient demographics (race, gender, age), duration, and type of treatment (chronic or acute phase, initiation, or maintenance treatment, early or late in therapy), use of scales and severity measures for side effect assessment, mode of clozapine dose titration (rapid or slow), etc. All these factors can impact differently adverse effect development, identification, and evaluation, thus influencing outcomes set in different trials. In most studies, there is a great preponderance of men over women, oftentimes higher than three-fold. Gender differences in terms of side effects is an underestimated field, and most studies, with the exception of Anderson et al. [74], do not stratify samples by sex. Age also presents a source of side effect variability, ranging from 21 to 75, with a mean value between 30 and 45 years in adult samples. This is an additional factor affecting side effect profiles since women and the elderly are generally more susceptible to side effects than men or younger people [96,97]. Race is sometimes not reported; in some studies, samples are uniform in terms of race while others have mixed samples, or race differs significantly between samples (i.e., an excess of patients are of a certain race). Notably, ethnic pharmacokinetic differences exist and should be taken into consideration [98]. Moreover, some side effects may be dependent on the duration of the treatment, for example, leukopenia and myocarditis or myocardiopathy are more frequent in the first months of therapy than later [31,99]. Nonetheless, these two studies reported higher rates of adverse effects with the escalation of clozapine plasma levels [40, 43]]. The rate of occurrence of adverse effects may also differ depending on the duration of clozapine exposure which also varied among studies. Moreover, the use of validated instruments for side effect identification and severity evaluation as well as, laboratory biomarkers used in some studies may result in more rigorous detection of adverse effects compared to patients’ self-reports or routine clinical interrogations used in others. We also noted that most studies were conducted in adults with only two studies in pediatric samples, thus limiting our understanding of the role of plasma clozapine levels on adverse effects in children.

It would be desirable to set specific thresholds for each type A ADRs, in order to facilitate an informed decision by the treating psychiatrist. However, from our research, it became apparent that different studies set different thresholds for the same side effect and these thresholds may vary significantly from one another. This could be due to the fact that different investigators employed different methodologies for plasma level determination (as summarized in Table 1) or collected samples at different time points from different populations under different comedications, as described in paragraph 2.1. As a result of this heterogeneity in plasma clozapine level measurements, it would be misleading and largely inaccurate to proceed with threshold recommendations.

To this date, trough plasma levels (C_trough_) of clozapine are used and almost all studies relating plasma clozapine levels to side effects use these metrics. Nonetheless, peak plasma levels, usually around 2–3 h after drug administration may also be valuable since they may relate to the severity of dose-dependent side effects. As a future direction, a more detailed pharmacokinetic evaluation that will include not only trough but peak levels may be more informative. In that case, C_trough_/AUC would be a handy parameter to determine and relate to ADR frequency and severity.

## 4. Materials and Methods

### 4.1. Protocol and Registration

The Preferred Reporting Items for Systematic Reviews and Meta-Analyses (PRISMA) statement recommendations were followed for background, search strategy, methods, results, discussion, and conclusions [100]. Ethical approval was not required for this systematic review since all included data have been previously published with ethical approval.

### 4.2. Eligibility Criteria

Studies included were conducted in humans, and article language was limited to English. The main screening criterion at all levels of our search was the identification of publications reporting data of clozapine side effects in relation to its plasma/serum levels. Original research papers and case reports were included. Review papers relevant to our main screening criterion were used as a source of additional original research papers by manually searching their reference lists. Studies that used experiments or animals, or did not provide information on side effects in relation to plasma clozapine levels, as well as letters reporting no primary data, conference abstracts, and articles that analyzed drug-drug interactions were excluded. ZD, CAK, and VK performed the literature review and collected all relevant studies. MS, EAK, PG, KEK, and OA participated in the independent appraisal of the collected manuscripts, article selection, and the evaluation of reported data.

### 4.3. Search Strategy and Study Selection

A literature search in SCOPUS and PubMed databases was conducted from inception to August 2021. In both databases, we used the terms ‘Clozapine Levels’, ‘Side Effects’, ‘Adverse Effects’, ‘Adverse Drug Reaction’, and ‘Toxicity’ in different combinations. No limitation or other filters were applied to the searches. Studies that fulfilled the inclusion criteria, after exclusion of duplicates, were screened at the title and abstract level, and then selected articles were screened at the full-text level.

### 4.4. Data Collection Process

Each publication was analyzed for its type (i.e., review, original paper, metanalysis, etc.), date of publication, names of authors and their affiliations, quality of evidence according to the Jadad scoring system [101], the country where the study took place, sample size, cohort status, sex, race, mean age, diagnosis, and diagnostic tools, the analytical technique used for clozapine plasma level determination, duration of clozapine administration, mean dose administered, time of clozapine level measurement (peak or trough), diagnoses and comedications, the reported plasma levels, the recorded side effects and the assessment tools for identifying side effects, and the main conclusions of each publication.

### 4.5. Outcomes

Side effects in relation to plasma clozapine levels were categorized and presented by the physiological organ system.

### 4.6. Evaluation of Study Quality Using the Jadad Scoring System

The Jadad score [101] was used for the assessment of the quality of evidence of each study as low or high. This is a simplified quality assessment tool that allows easy and quick evaluation of the quality of clinical evidence. Briefly, the assessment was based on the type and protocol of randomization and blinding procedures and the description and accountability of withdrawals and dropouts. Studies with a score equal to or lower than 2 were classified as low quality, while studies with a score equal to or greater than 3 were characterized as high quality. The classification of evidence based on the Jadad score is briefly described in Table 9.

## 5. Conclusions

In conclusion, monitoring clozapine and its metabolites plasma levels is a reasonable strategy for personalization of treatment, improvement of drug safety, and enhancement of tolerance, in TRS patients. This may further contribute to overcoming the underutilization of clozapine, a drug with superior antipsychotic efficacy and the only effective in TRS. There is plenty of room for more and better-designed studies, emphasizing more the influences of gender, age, race, specific diagnoses, comorbidities, genomics, and other factors. Further exploring the intriguing interrelationship of clozapine plasma levels with inflammation, and the effects of the drug on the cardiac muscle, the autonomous and endocrine systems, the gastrointestinal tract, and energy metabolism, are important directions for future research.

## Figures and Tables

**Table 1:** Methods of plasma clozapine measurement.

Methods for Measurement of Plasma CLOZ Levels	References
High-Performance Liquid Chromatography (HPLC)	Combs et al. [36], Carceller-Sindreu et al. [68], Olesen et al. [35], Oyewumi et al. [37], Hummer et al. [69], Rajii et al. [59], Wong et al. [42], Oyewumi et al. [64], Melkersson et al. [49], Yusufi et al. [56], Spina et al. [46], Melkersson and Dahl [70], Melkersson et al. [45], Lu et al. [71], Rechlin et al. [72], Eschweiler et al. [73], Mauri et al. [38], Vaquero-Baez et al. [67], Anderson et al. [74]
Reversed-Phase High-Performance Liquid Chromatography (RP-HPLC)	Haring et al. [58], Lin et al. [60], Shen et al. [55]
Liquid Chromatography (LC)	Seppala et al. [75], Kim et al. [47]
Reversed-Phase Liquid Chromatography	Centorrino et al. [41], Frazier et al. [43]
Gas Chromatography (GC)	Ackenheil [44], De Leon et al. [66], De Leon et al. [62], De Leon et al. [63], Freudenreich et al. [34], VanderZwaag et al. [39]
Liquid Chromatography coupled with Mass Spectrometry (LC-MS)	Curto et al. [76], Vasudev et al. [51]
Liquid Chromatography coupled with tandem Mass Spectrometry (LC-MS/MS)	Gharab et al. [77], Smith et al. [78]
Not specified	Hummer et al. [52], Every-Palmer et al. [53], Grande et al. [79], Sporn et al. [40], Khan et al. [80], Subramaniam et al. [65], Lally et al. [81], Nilsson et al. [48], Meltzer et al. [50], Bailey et al. [54], Hummer et al. [57]

**Table 2:** General scores of adverse effect scales or combinations of adverse effects in relation to clozapine plasma levels.

Reference	Type of Study	Age (Mean) (Years)	Comedication	Averaged Serum Levels of Clozapine (ng/mL)	Duration of CLOZ Exposure	Reported Side Effects	Correlation to CLOZ Plasma Levels	Jadad Score
[56]	Cross-sectional	39.3 ± 8.8	Mood stabilizer (31%), Anticholinergic (18%), antidepressant (16%), other antipsychotic (5%), anxiolytic or hypnotic (5%)	CLOZ: 530 ± 370, NCLOZ: 310 ± 190	Median (range): 30 (3–156) months	Parkinsonism, akathisia, tardive dyskinesia, non-neurological side effects including cardiovascular, gastrointestinal, sexual genitourinary and others	CLOZ levels vs. total ANSSERS score: r = 0.29, *p* < 0.004 CLOZ levels vs. moderate and severe ADRs: r = 0.23, *p* < 0.03	1
[43]	Open-label	13.3 ± 2.7 (range 9–16)	No concomitant medications	Crude (ng/mL): 289 ± 116, Normalized (ng/mL-mg-Kg): 99 ± 37.3	6 weeks	Adverse effects included sedation, enuresis, tachycardia, sialorrhea, reduced neutrophil count, increased hepatic transaminases	Moderate and severe side effects vs. CLOZ + NCLOZ: r = 0.4, *p* = 0.002) Moderate and severe side effects vs. NCLOZ: r = 0.6, *p* = 0.002 Moderate and severe side effects vs. CLOZ + NCLOZ + NOX levels: r = 0.4, *p* = 0.03	0
[46]	Prospective, open follow-up	19–65	No	CLOZ: 385 ± 183 (range 147–974) NCLOZ: 174 ± 84 (range: 43–445)	12 weeks	Hypersalivation, constipation, tachycardia, dizziness, sedation, weight gain	No	1
[41]	Cross-sectional	36.6 ± 9.1 (range 20–54)	Benzodiazepines, lithium, antidepressants	CLOZ: mean = 297 (median: 291), among 68 samples. Subsample not exposed to fluoxetine or valproate (*n* = 27): 239 ± 159	2.15 ± 2.30 years	SAFTEE scale ADRs	No	0

CLOZ: clozapine, NCLOZ: norclozapine, ANNSERS: Antipsychotic Non-Neurological Side Effects Rating Scale, SAFTEE: Systematic Assessment for Treatment Emergent Effects.

**Table 3:** Nervous system and psychiatric adverse effects in relation to CLOZ plasma levels.

Reference	Type of Study	Age (Mean) (Years)	Comedication	Averaged Serum Levels of Clozapine (ng/mL)	Duration	Reported Side Effects	Correlation to Clozapine Plasma Levels	Jadad Score
[34]	Prospective, randomized	38 (range: 21–56)	Rarely given doses of haloperidol or fluphenazine	group I (*n* = 16): 50–150 group II (*n* = 22): 200–300 group III (*n* = 12): 350–450	12 weeks	Seizures, EEG changes, sleepiness	EEG abnormalities, more severe than borderline, rate: group III: 73% vs. group I: (20%) and group II: 21% (*p* = 0.006) Severity: group I 0.9 ± 1.8, group II 1.0 ± 1.5, group III 3.4 ± 1.9 (*p* < 0.001) Spike/sharp activity: no correlation Slowing: more slowing in group III vs. group II and I (*p* = 0.049), positive correlation with levels (r = 0.44, *p* = 0.002), possible cutoff: 300 ng/mL Sleepiness: positive correlation with levels (r = 0.33, *p* = 0.029). The EEG slowing correlated with observed sleepiness	2
[58]	Prospective, observational	31.7 ± 10.2	No	Whole sample (*n* = 29): 161.3 ± 150.0 group 1 (*n* = 14): 81.6 ± 64.6, group 2 (*n* = 15): 235.7 ± 169.8	20.2 ± 16.8 days	EEG changes	Plasma levels significantly different among groups according to severity of EEG changes. Group 1 (*n* = 14): degree 0–1, plasma levels: 81.6 ± 64.6 ng/mL (95% CI = 44.3–118.9). Group 2 (*n* = 15): degree 2–4, plasma levels: 235.7 ± 169.8 mg.mL (95% CI: 141.7–329.7) (*p* = 0.0009)	0
[35]	Cross-sectional, blinded for EEG measures	37.6 ± 1.67	Levomepromazine or chlorprothixene for sedation up to 100 mg/day. Other medication as usual	Median CLOZ: 351 (231–615) (range: 64–1824)	2.5 (1.0–9.0) years	EEG changes	Severity correlated to plasma CLOZ (r = 0.43; *p* < 0.05) but not NCLOZ levels. CLOZ concentrations ≥1306 ng/mL lead to progressive gradual EEG changes	0
[39]	Prospective, randomized, double-blind	3 (range 21–56)	All psychoactive medication tapered off. Valproate in two patients with a history of seizures	Low: 91 ± 15 (50–150), medium: 251 ± 13 (200–300), high: 396 ± 16 (350–450)	12 weeks	Sleepiness, EPS	Trend of sleepiness and serum level to correlate at week 6 (*p* = 0.08), but no significance at week 12. EPS improved over time, with no group-by-time interactions.	2
[40]	Prospective, longitudinal, observational, partly double-blind (*n* = 22) partly open-label (*n* = 32)	Range: 8–18	No	Week 6: CLOZ = 455 ± 285.1, NCLOZ + CLOZ 302.4 ± 142.2	6 week treatment at first and then 2–6 years follow-up	EEG changes seizures, akathisia	Rates of side effects were not directly associated with CLOZ or nor CLOZ blood levels or their ratio	0
[41]	Cross-sectional	36.6 ± 9.1 (range 20–54)	Benzodiazepines, lithium, antidepressants, other medically indicated agents	CLOZ: mean = 297 (median: 291), among 68 samples. Subsample not exposed to fluoxetine or valproate (*n* = 27): 239 ± 159	2.15 ± 2.30 years	Sedation	No	0
[42]	Prospective, non-randomized, double-blind, observational	37.61 ± 8.68 (range 21–63)	Chloral hydrate, Lorazepam, Valproate	Week 6: cloz: 470.20 ± 234.2, range 100–1220 norclozapine: 233.06 ± 105.56, range 70–670, Week 12: cloz 681 ± 390.71, range 220–1920, NCLOZ: 297.8 ± 146.49 range 8–720	12 weeks	EEG changes, akathisia, EPS	No significant correlation between plasma levels and EEG abnormalities on week 6, BARS, AIMS, SAS scores on weeks 6 and 12, negative correlation with sedation at week 6, which was clinically implausible	0
[47]	Retrospective, observational	37.7 ± 11.7	Valproate (*n* = 25, 35.2%) Mood stabilizers (lamotrigine, valproate, lithium, topiramate) (*n* = 31) Antipsychotics, antidepressants, benzodiazepines, or mood stabilizers in combination (*n* = 68)	CLOZ: 429.4 ± 264.1 NCLOZ: 197.8 ± 132.6	4.6 ± 4.9 years	EEG changes	Positive correlation with CLOZ levels (*p* = 0.008). No correlation with NCLOZ levels (*p* = 0.12). Patients with CLOZ levels > 600 ng/mL had higher rate of EEG abnormalities (93.8%) than those with levels <600 ng/mL (65.5%) (*p* = 0.02)	0
[75]	Cross-sectional	Age: 42.5 (range: 20–65)	Clozapine monotherapy: 65.4%, CLOZ + atypical: 22.5%, ClOZ + typical: 9.7%, CLOZ + typical + atypical: 1.7%	Men: 722 ± 366, Women: 886 ± 480 (*p* = 0.03) Total sample: 778 ± 444.57	3–12 months (1.7%), 1–5 years (32.5%), 5 years (57.8%), not specified (8%)	Difficulty in concentrating, tension, difficulty remembering things, depression, restlessness, difficulty getting to sleep	Yes, only with the Depression/Anxiety score	0
[68]	Single-blind, cross-sectional	Group I: 45 ± 10.3, Group II 47.2 ± 7.5	No	Group I: CLOZ ≥300 Group II: CLOZ <300	≥5 years	Cognitive performance	No relationship between clozapine plasma levels and cognitive performance. Tendency to significance regarding the executive test (31% of variability of number of attempts in the WCST was explained by clozapine plasma levels)	0
[43]	Open-label	13.3 ± 2.7 (range 9–16)	No	Crude (ng/mL): 289 ± 116, normalized (ng/mL-mg-Kg): 99 ± 37.3	6 weeks	Sedation	No	0
[59]	Retrospective analysis of clinically collected cross-sectional data.	41.6 ± 12.0	Not reported	All subjects (*n* = 73): 458.5 ± 248.8 Low cognitive impairment (*n* = 57): 437.1 ± 249.6 High cognitive impairment (*n* = 16): 534.7 ± 237.7	≥3 months	Cognitive impairment	Sixteen subjects (21.9%) had high cognitive impairment and the rest had low cognitive impairment. Age and clozapine levels were associated with high cognitive impairment, as well as clozapine/desmethylclozapine raitio (OR: 7.3). Yes	0
[56]	Cross-sectional	39.3 ± 8.8	Mood stabilizer (31%), Anticholinergic (18%), antidepressant (16%) other antipsychotic (5%), anxiolytic or hypnotic (5%)	CLOZ: 530 ± 370, NCLOZ: 310 ± 190	Median (range): 30 (3–156) months	Memory and concentration problems, night-time sleep problems, Parkinsonism, akathisia, tardive dyskinesia	No correlation	1
[62]	Double-blind, prospective. Randomized to clozapine doses	Not reported	Haloperidol	End of first trial (16 weeks): 335 ± 340	First trial: 16 weeks, second trial:16 weeks	Drowsiness, sedation	Clozapine levels were very good predictors of serum antimuscarinic activity in doses of 300 mg/d or higher. Sedation showed no significant association with serum antimuscarinic activity.	2
[60]	Retrospective, naturalistic, 1-year study/cross-sectional	37.9 ± 9.3	Fluvoxamine (*n* = 8), fluoxetine (*n* = 2), sertraline (*n* = 2), paroxetine (*n* = 1), valproate (*n* = 15)	Patients with OCS: 595.1 ± 364.9 (range 84–1491) Patients without OCS: 433.5 ± 252.8 (range 82–1273)	Patients with OCS: 81.8 ± 32.2 months, Patients without OCS: 56.1 ± 40.6 months	OCS	Plasma concentration of clozapine was significantly higher in patients with OCS than in those without (*p* = 0.001)	0

CLOZ: clozapine, NCLOZ: norclozapine, OCS: obsessive-compulsive symptoms.

**Table 4:** Cardiovascular adverse effects in relation to CLOZAPINE blood levels.

Reference	Type of Study	Age (Mean)	Comedication	Averaged Serum Levels of Clozapine (ng/mL)	Duration	Reported Side Effects	Correlation to Clozapine Plasma Level	Jadad Score
[35]	Cross-sectional	37.6 ± 1.67	Levomepromazine or chlorprothixene. Other medication as usual	Median S-CLOZ: 351 (231–615) (range: 64–1824)	2.5 (1.0–9.0) years	Increased pulse rate (>80 bpm), tachycardia (>100 bpm), orthostatic hypotension	No	0
[39]	Prospective, randomized, double-blind	38 (range 21–56)	Haloperidol, phluphenazine, valproate	Low: 91 ± 15 (50–150), medium: 251 ± 13 (200–300), high: 396 ± 16 (350–450)	12 weeks	Tachycardia: Orthostaic hypotension:	No	2
[40]	Prospective, longitudinal, observational, partly double-blind (*n* = 22) partly open-label (*n* = 32)	Range: 8–18	No	Week 6: CLOZ 455 ± 285.1, NCLOZ 302.4 ± 142.2	6 week treatment at first and then 2–6 years follow-up	Hypertension (>140/90 mmHg), orthostatic hypotension, tachycardia (>120 bpm)	No	0
[42]	Prospective, non-randomized, double-blind, observational	37.61 ± 8.68 (range 21–63)	Chloral hydrate. Lorazepam. Valproate	Week 6: Clozapine: 470.20 ± 234.23, range 100–1220 Norclozapine: 233.06 ± 105.56, range 70–670, Week 12: clozapine 681 ± 390.71 ng/mL (range 220–1920 ng/mL), NCLOZ 297.8 ± 146.49 ng/mL (range 8–720 ng/mL)	12 weeks	QTc alterations	No	0
[44]	Prospective, open-label	Not reported	Not reported	200–300 ng/mL (divided into three groups I: <30 ng/mL, II: 30–100 ng/mL, III: >100 ng/mL for paranoid-hallucinatory schizophrenic patients and manic syndrome)	30 days	Orthostatic hypontension, temperature	Yes, with severity of orthostatic dysregulation	0
[43]	Open-label	13.3 ± 2.7 (range 9–16)	No	Crude (ng/mL): 289 ± 116, Normalized (ng/mL-mg-Kg): 99 ± 37.3	6 weeks	Tachycardia	No	0
[64]	Prospective longitudinal	Not reported	Lorazepam allowed	Mean serum CLOZ level by week (1–8): 63.5 ± 46.0 199.3 ± 149.1 251.9 ± 178.0 300.7 ± 200.2 316.2 ± 189.4 364.7 ± 195.6 351.9 ± 176.2 379.5 ± 156.5	First 8 weeks of administration	Hypotension, temperature, pulse	The blood pressure and pulse did not change significantly from baseline to week 8. Temperature was inversely related to clozapine dose (*p* < 0.003) Higher norclozapine to clozapine ratios were associated with higher BP measures (*p* = 0.002). The magnitude of these relationships is weak (r < 0.30). There is a tendency to autonomic dysregulation during clozapine use	0
[80]	Cohort, prospective study, open	44 ± 12	Not reported	475 ± 236	Mean follow-up of 9 ± 6 years	Myocarditis, sudden death	No	0
[79]	Retrospective, review of case records	31.21 ± 9.59	None: 52.44% Antidepressants: 19.51% Mood stabilizers:31.71% Stimulants: 1.22%	CLOZ: 305.56 ± 299.64, nor CLZ: 160.23 ± 105.12	18 weeks	-	No	1
[76]	Preliminary prospective study	35.5 ± 11.0	One other antipsychotic, one mood-stabilizing drug, or a benzodiazepine	CLOZ: 124 ± 70.8 nor CLOZ: 52.3 ± 35.7	4 weeks	Increased Heart Rate, Myocardial Performance Index (MPI) > 0.44. Reduced LV functioning	No	0
[72]	Cross-sectional	Patients: 40.7 (range: 21–68) Controls: 41.2 (range: 20–64)	No	290	>8 weeks	HRV	Yes, negative (inverse) correlation	0
[73]	Retrospective	42.1 (range: 19–73)	Haloperidol, benzodiazepines, SSRIs, Carbamazepine	331 ± 294 (range: 65–1475)	>1 week	HRV	Yes, negative (inverse) correlation	0
[48]	Cross-sectional	33.5 (range: 26–41)	Not reported	451 (range: 337–569) (*n* = 29)	7 (range: 3–13)	Persistent tachycardia	No	0

HRV: Heart Rate Variability.

**Table 5:** Metabolic and endocrine adverse effects in relation to clozapine plasma levels.

Reference	Type of Study	Age (Mean) (Years)	Comedication	Averaged Serum Levels of Clozapine (ng/mL)	Duration	Reported Side Effects	Correlation to Clozapine Plasma Levels	Jadad Score
[63]	Prospective, double-blind, randomized	44.8 ± 9.6	No	CLOZ: >350 in responders	29.7 ± 13.2 weeks (16 weeks, 32 weeks, 48 weeks, based on response status)	Weight gain	In nonsmokers (*n* = 8) r = 0.89 (*p* = 0.046)	2
[81]	Prospective, observational, open-label	37.4 ± 9.3, (range 22–57)	Not reported	500 ± 280 (range 70–1360)	32.6 ± 6.6 weeks (23–62 weeks)	Weight gain, waist circumference	No (r = −0.04, *p* = 0.77) at follow-up	0
[65]	Cross-sectional	38.2 ± 11.3 (range 22–74); Caucasians: 40.2 ± 8.6, Asians: 36.3 ± 13.4	Not reported	Caucasians: 415.3 ± 185.8 Asians: 417.1 ± 290.8	≥6 months	Lipid profiles, fasting glucose levels	No	0
[70]	Cross-sectional	46 (29–63)	Not reported	Median: 359.1 (range 60.5–810.46)	At least 6 months Median: 5.3 years (range 0.5–16.3 years)	Fasting insulin, C-peptide, insulin-like growth factor I, insulin-like growth factor binding protein-1, leptin, glucose and lipids	CLOZ vs. insulin: r = 0.51, *p* = 0.03 CLOZ vs. C-peptide: r = 0.48, *p* = 0.04 CLOZ vs. triglycerides: r = 0.50, *p* = 0.03)	0
[49]	Cross-sectional	35 (26–47)	No	28.8–721	2.7 (range 0.5–7.3 years)	Fasting glucose, insulin, growth hormone (GH)-dependent insulin-like growth factor I (IGF-I), and insulin-dependent insulin-like growth factor binding protein-1	CLOZ vs. insulin levels	0
[50]	Open, prospective	Males: 34.7 ± 8.1 Females: 36.2 ± 11.6	Benztropine (*n* = 6), diphenylhydantoin (*n* = 2), fluoxetine (*n* = 1), divalproate (*n* = 1)	6 weeks: 388 ± 242 6 months: 444 ± 355	6 months	Weight gain	No	0
[51]	Cross-sectional	36.5 ± 11.3	Antipsychotics (61.9%), antidepressants (14.3%), mood stabilizers (21.4%)	CLOZ: 1613.57 ± 976.05 NCLOZ: 964.60 ± 976.05	Stable clozapine therapy for at least 6 months	Metabolic syndrome BMI	CLOZ vs. metabolic syndrome	0
[74]	Retrospective	Males: 36.9 (95% CI: 33.9–39.8) Females: 39 (95% CI: 35.4–42.7)	Aripiprazole (*n* = 6) Amisulpride (*n* = 4) Haloperidol (*n* = 2) For the females: estrogen-containing contraceptive pill (*n* = 5), estrogen-containing hormone replacement treatment	CLOZ: Males: 440 (10th–90th percentile: 260–700) Females: 490 (10th–90th percentile: 270–790) NCLOZ: Males: 310 (10th–90th percentile: 260–350) Females: 310 (10th–90th percentile: 270–340	Males: 4.4 (95% CI: 1.2–10.3) years Females: 5.1 (95% CI: 2.3–7.9) years	BMI Fasting blood glucose HDL	CLOZ vs. BMI CLOZ vs. fasting blood glucose	0
[45]	Cross-sectional	Median: 41 (range: 29–36)	Benzodiazepines (*n* = 4), and/or levomepromazine (*n* = 3) and/or lithium (*n* = 1)	CLOZ: 392(69–918) NCLOZ: 288 (88–641)	6.9 years (range: 0.7–16.3 years)	Elevated blood glucose, elevated levels of insulin, elevated levels of C-peptide, elevated triglycerides, cholesterol, HOMA-IR	CLOZ vs. insulin: r = 0.53, *p* = 0.03, CLOZ vs. C-peptide r = 0.51, *p* = 0.04 CLOZ vs. triglyceride levels: r = 0.46, *p* = 0.06	0
[71]	Prospective, randomized	Coadministration group: 32.9 ± 8.5 Monotherapy group: 35.1 ± 9.4	No	Coadministration group: CLOZ: 509.8 ± 281.1 NCLOZ: 179.0 ± 95.8 Monotherapy group: CLOZ: 502.0 ± 220.6 NCLOZ: 242.8 ± 100.3	12 weeks	Serum glucose, cholesterol, and TRG levels, weight gain	NCLOZ vs. weight gain: r = 0.27, *p* = 0.026 NCLOZ vs. blood sugar: r = 0.34, *p* = 0.005 NCLOZ vs. Triglycerides: r = 0.27, *p* = 0.028	2
[77]	Cross-sectional, controlled	Patients: 40.94 ± 10.15 Controls: 40.09 ± 1.67	Not reported	CLOZ: 594.90 ± 492.90 NCLOZ: 220.33 ± 182.55	At least 4 months	Blood measures	CLOZ vs. total cholesterol: r = 0.34, *p* = 0.04	0
[41]	Cross-sectional	36.6 ± 9.1 (range 20–54)	Benzodiazepines, lithium, antidepressants, other medically indicated agents	CLOZ: mean = 297 (median: 291), among 68 samples. Subsample not exposed to fluoxetine or valproate (*n* = 27): 239 ± 159	2.15 ± 2.30 years	Excess weight	No	0
[66]	Double-blind dose–response	49 females (32–60 years old), 42 males (31–58 years old)	No	400–1600	16-week	Hyperprolactinemia	For every 100 ng/mL increase in plasma clozapine levels, average increments in prolactin levels of 0.45 ng/mL in females and 0.15 ng/mL in males were recorded	2
[75]	Cross-sectional	42.5 (20–65)	CLOZ Monotherapy: 65.4%, CLOZ + atypical: 22.5%, CLOZ + typical: 9.7%, CLOZ +typical + atypical: 1.7%	Men (722 ± 366) and women (886 ± 480)	3–12 months (1.7%), 1–5 years (32.5%). 5 years (57.8%), unspecified (8%)	Menstrual problems	No correlation with CLOZ + NCLOZ concentration	1
[77]	Cross-sectional, controlled	Patients: 40.94 ± 10.15 Controls: 40.09 ± 1.67	Not reported	594.90 ± 492.90 220.33 ± 182.55	At least 4 months	Blood measures	No correlation with TSH, FT4, PRL. CLOZ vs. FT3: r = −0.373, *p* = 0.021	0

**Table 6:** Gastrointestinal adverse effects in relation to CLOZAPINE blood levels.

Reference	Type of Study	Age (Mean) (Years)	Comedication	Averaged Serum Levels of Clozapine (ng/mL)	Duration	Reported Side Effects	Correlation to Clozapine Plasma Levels	Jadad Score
[41]	Cross-sectional	36.6 ± 9.1 (range 20–54)	Benzodiazepines, lithium, antidepressants, other medically indicated agents	Median 291, range: 15–726	2.15 ± 2.30 years	Nocturnal sialorrhea, drooling, parotid swelling, constipation	No	0
[46]	Prospective, observational follow-up	19–65	Benzodiazepines	CLOZ: 385 ± 183 (range 147–974) NCLOZ: 174 ± 84 (range: 43–445)	12 weeks	Hypersalivation, constipation	No	1
[52]	Prospective	CLOZAPINE subsample: 31.37 ± 11.8	Not reported	165.4 ± 163.4	18 weeks	Pathologic liver function tests (LFTs): SGOT, SGPT, GGT, ALP, bilirubin	Yes, for SGPT only	0
[35]	Cross-sectional, naturalistic	Range 22–55	Nortriptyline, levomepromazine, clonazepam, hyoscyamine, oxazepam, chlorprothixene, phenobarbital, nitrazepam, biperiden, orphenadrine, benztropine, diazepam, piroxicam, disulfiram	Median: 1076 (range 706–1882) NCLOZ/CLOZ ratio: 0.77 ± 0.17	Median: 2.5 (range 1.0–9.0) years	Increased liver enzyme activity (increased GGT, ALP, AST, ALT)	No	0
[77]	Cross-sectional, controlled	Patients: 40.94 ± 10.15 Controls: 40.09 ± 1.67	Not reported	594.90 ± 492.90 220.33 ± 182.55	At least 4 months	AST, ALT	No	
[65]	Cross-sectional	38.2 ± 11.3 (range 22–74); Caucasians: 40.2 ± 8.6, Asians: 36.3 ± 13.4	Not reported	Caucasians: 415.3 ± 185.8, Asians: 417.1 ± 290.8	≥6 months	Elevated levels of alanine (ALT) and aspartate (AST) transferases	No	0
[43]	Open-label trial	13.3 ± 2.7 (range 9–16)	No	289 ± 116	6 weeks	Increased hepatic transaminase	No	0
[40]	Data from double-blind and open-label clozapine trials	13.5 ± 2.5 (range 7.0–19.1)	Not reported	455.6 ± 285.1 (*n* = 46)	6 weeks	Elevated liver enzymes (AST, ALP, ALT)	No	0
[53]	Cross-sectional	39.3 ± 9.8 (range 20–61) (CLOZAPINE group: 37 ± 8.2, NON CLOZAPINE group:	Laxatives (laxsol, polyethylene glycol, lactulose), antipsychotics (risperidone, aripiprazole, haloperidol, amisulpride, aripiprazole + quetiapine) omeprazole, metformin, cholecalciferol	489 ± 137 (range 284–885)	At least 3 months	Colonic hypomotility	Positive correlation Clear colonic hypomotility in 80% of CLOZAPINE patients, with Colonic Transit Time (CCT) four times longer than population morms (*p* < 0.0001) and NON-CLOZAPINE patients (*p* < 0.0001)	0
[54]	Retrospective, collection of records	Males: 43.5 ± 10.1 Females: 47.2 ± 11.2	Anticholinergic agents	CLOZ: Laxative users: 533 ± 0.29 Non-laxative users: 486 ± 30 NCLOZ: Laxative users: 337 ± 0.19 Non-laxative users: 269 ± 0.18	>3 months	Constipation	NCLOZ vs. laxative use, *p* = 0.046)	0

**Table 7:** Studies reporting hematological, genitourinary, and other adverse effects in relation to CLOZAPINE blood levels.

Reference	Type of Study	Age (Mean) (Years)	Comedication	Averaged Serum Levels of Clozapine (ng/mL)	Duration	Reported Side Effects	Correlation to Clozapine Plasma Levels	Jadad Score
**Hematological System**								
[41]	Cross-sectional	35.6 ± 9.3 (range 17–54)	Not reported	CLOZ: 304 ± 174 NCLOZ: 216 ± 133	2.16 ± 0.35 years	WBC Neutrophil Count	No	0
[35]	Cross-sectional	37.6 ± 1.67	Levomepromazine or chlorprothixene	Median 430.4 (282.4–752.8)	At least 3 months (range: 1–17 years)	Mild leukocytosis and increased erythrocyte folate	No	0
[37]	Prospective longitudinal	35.2 ± 10.2 (range: 18–57)	Lorazepam	379.5 ± 156	4 to 8 weeks	Hematological parameters (WBC, red blood count, neutrophils, platelets, and lymphocytes counts or hemoglobin and hematocrit)	CLOZ vs. platelets count r = 0.32, *p* = 0.042	0
[40]	Prospective, double-blind (*n* = 22), open-label (*n* = 32)	13.5 ± 2.5 (range 7.0–19.1)	Mood stabilizers and antidepressants	CLOZ: 455 ± 285.1 NCLOZ: 302.4 ± 142.2	6 week treatment (2–6 years follow-up)	Neutropenia	No	0
[43]	Prospective, open-label/double-blind	13.3 ± 2.7 (range 9–16)	No	CLOZ: 289 ± 116 NCLOZ: 410 ± 190	6 weeks	Moderate neutropenia	No	0
[36]	Retrospective chart review	36.4 ± 10.4	Not reported	CLOZ: 389 ± 386 NCLOZ: 199 ± 216	Mean duration not reported; samples for measuring drug levels drawn at least once during the first 3 months and then randomly after	WBC or granulocyte counts	No	0
[78]	Retrospective, observational	34 (median) 20–84 (range)	Not reported	CLOZ: 1068 (26–3955) NCLOZ: 712 (31–5813)	Not reported	Absolute neutrophil count (ANC)	NCLOZ vs. ANC *p* = 0.002)	0
[77]	Cross-sectional, controlled	Patients: 40.94 ± 10.15 Controls: 40.09 ± 1.67	Not reported	594.90 ± 492.90 220.33 ± 182.55	At least 4 months	Hematological parameters	NCLOZ vs. PLT: r = −0.362, *p* = 0.025 NCLOZ vs. HGB: r = −0.342, *p* = 0.025, NCLOZ vs. NEU: r = −0.385, *p* = 0.017	0
[38]	Prospective, open-label	34.62 ± 7.56 (range: 25–48)	No	CLOZ: 266.27 ± 197.44 (25–1270) NCLOZ: 169.0 ± 127.94 (25–1280)	9 weeks	Leucocyte count Neutrophil count	CLOZ vs. NEU: r = 026, *p* = 0.001 NCLOZ vs. NEU r = 0.20, *p* = 0.01 .NCLOZ/CLOZ ratio vs. NEU: r = −0.26, *p* = 0.002	0
[69]	Prospective, open	Males: 28.9 ± 9.7 Females: 34.2 ± 10.7	Not reported	145.8 ± 160.1 (3.1–1571.0)	16.7 ± 24.7 weeks	WBC disorders: Transient neutropenia, eosinophilia, and leukocytosis. Progressive neutropenia, chronic leukocytosis	No	0
[67]	Cross-sectional	Males: 34.68 ± 1.58 Females: 31.58 ± 1.29	Clonazepam, Fluoxetine, Paroxetine, Lithium Mirtazapine, Metformin, Sulpiride, valproate, venlafaxine, Duloxetine, escitalopram, imipramine, losartan, omeprazole, pregabalin	Males: 290.11 ± 51.56 Females: 336.36 ± 29.16	Males: 10.05 ± 1.74 months Females: 6.63 ± 1.15 months	Leukocyte count Neutrophil count	CLOZ vs. NEU: r: 0.631, *p* = 0.004 CLOZ vs. leukocyte count r: −0.725, *p* = 0.001	0
**Geniturinary Side Effects**								
[62]	Prospective, double-blind	Not reported	Not reported	CLOZ: 325 ± 199 NCLOZ: 576 ± 326 (dose-dependent)	16 weeks	Urinary disturbances	No	2
[40]	Prospective, double-blind (*n* = 22), open-label (*n* = 32)	13.5 ± 2.5 (range 7.0–19.1)	Mood stabilizers and antidepressants	CLOZ: 455 ± 285.1 NCLOZ: 302.4 ± 142.2	6 week treatment (2–6 years follow-up)	Enuresis	No	0
[43]	Prospective, open-label/double-blind	13.3 ± 2.7 (range 9–16)	No	CLOZ: 289 ± 116 NCLOZ: 410 ± 190	6 weeks	Enuresis	No	0
[41]	Cross-sectional	35.6 ± 9.3 (range 17–54)	Not reported	CLOZ: 304 ± 174 NCLOZ: 216 ± 133	2.16 ± 0.35 years	Nocturnal enuresis	No	0
[57]	Prospective	28.6 ± 9.5	Benzodiazepines, anticholinergic drugs, b-blockers, antidepressants, anticonvulsants	183.3 ± 150.2	18 weeks	Sexual disturbances	CLOZ vs. sexual desire (*p* = 0.02) CLOZ and sexual functional disturbances (*p* = 0.008)	0
**Other**								
[55]	Clinical study	Group I: 38.8 (23–58), group II: 41.5 (21–58), group III: 43.9 (22–60)	Not mentioned	221.4 ± 109.6	38.3 ± 6.3 days (CLOZ group)	Serum anticardiolipin antibodies (aCL) IgM, IgG	CLOZ vs. aCL IgM: r = 0.461, *p* = 0.001	1
[77]	Cross-sectional, controlled	Patients: 40.94 ± 10.15 Controls: 40.09 ± 1.67	Not reported	CLOZ: 594.90 ± 4 NCLOZ: 92.90 220.33 ± 182.55	At least 4 months	B12, Na, K, ure, cre	No	0

**Table 8:** Classification of side effects according to their relationship to plasma clozapine or norclozapine levels, based on current evidence.

System	Side Effect		
	Positive Correlation with Plasma Clozapine Levels	No Significant Correlation with Plasma Clozapine Levels	Controversial
**Nervous system**	Εlectroencephalographic abnormalities, EEG slowing		Seizures, sedation
			Impaired cognitive performance (incl. memory problems and lack of concentration)
		Extrapyramidal symptoms and tardive dyskinesia	
**Psychiatric**	Obsessive-compulsive symptoms		
	Depression/anxiety factor score (incl. difficulty in concentrating, tension, difficulty remembering things, depression, restlessness, difficulty getting to sleep)		
**Cardiovascular**	Heart rate variability	QTc prolongation	Myocarditis, pericarditis
		Μyocardial function	
		Sudden death	
**Autonomic Dysregulation**	Body temperature dysregulation	Hypertension	Orthostatic hypotension
		Τachycardia	
**Metabolic**	Hyperinsulinemia, C-peptide	Weight gain	
	Hyperlipidemia	Hyperglycemia	
**Endocrine**	Inverse correlation with fT3	Hyperprolactinemia	
**Gastrointestinal**	Paralytic ileus, constipation, colonic hypomotilityElevation of serum liver enzymes	Sialorrhea, drooling	
**Geniturinary**		Enuresis	Sexual disturbances
**Other**	Elevation of IgM anticardiolipin antibodies		

**Table 9:** The Jadad scoring system and the interpretation of each score.

Question	Yes/No
Was the study described as random?	1/0
Was the randomization scheme described and appropriate?	1/0
Was the study described as double-blind?	1/0
Was the method of double-blinding appropriate?	1/0
Was there a description of dropouts and withdrawals?	1/0
**Quality Assessment Based on Total Score**	
**Jadad Score**	**Quality of evidence**
0–2	Low
3–5	High

## Data Availability

Not applicable.

## Supplementary Materials

The following supporting information can be downloaded at: https://www.mdpi.com/article/10.3390/ph15070817/s1, Table S1A: Case Reports referring to adverse effects of clozapine in relation to drug levels; Tables S1B–S7: Additional data from all included studies (cited in the main manuscript). References [90,102,103,104,105,106,107,108,109,110,111,112,113] are cited in the supplementary materials.
