# Supplementary material for: Adverse Drug Reactions in Relation to Clozapine Plasma Levels: A Systematic Review
Source: Pharmaceuticals (Basel). 2022 Jul 1;15(7):817. doi: 10.3390/ph15070817 (PMC9317288; doi:10.3390/ph15070817)
Supplement: Supplementary file 1 [file pharmaceuticals-15-00817-s001.zip › pharmaceuticals-1671577-supplementary-FC.pdf]

Systematic Review

Adverse Drug Reactions in Relation to Clozapine Plasma Levels: A Systematic Review

Maria Skokou [1,†], Eleni A. Karavia [2,†], Zoi Drakou [1,2,†], Vassiliki Konstantinopoulou [2,†], Christina-Anna Kavakioti [1], Philippos Gourzis [1], Kyriakos E. Kypreos [2,3] and Ourania Andreopoulou [1,*]

[1] Department of Psychiatry, General University Hospital of Patras, School of Medicine, University of Patras, Patras 26504, Greece; mskokou@upatras.gr (M.S.); zoidrakou@gmail.com (Z.D.); christiana-kav@hotmail.com (C.-A.K.); pgourzis@upatras.gr (P.G.)

[2] Department of Pharmacology, School of Medicine, University of Patras, Patras 26504, Greece; karaviae@hotmail.com (E.A.K.); venia45@yahoo.gr (V.K.); kkypreos@upatras.gr (K.E.K.)

[3] Department of Life Sciences, School of Sciences, European University of Cyprus, Nicosia 2404, Cyprus

* Correspondence: andreop@upatras.gr

† These authors contributed equally to this work.

Supplemental Data

Table S1A. Case Reports referring to adverse effects of clozapine in relation to drug levels.

Reference	Location	Patients	Age	Race	Diagnosis	Comedication	Serum levels of clozapine (ng/ml)	Duration of treatment	Reported Side effects
[102]	USA	2, females	24/ 47	Not reported	schizophrenia	None	Case 1: 1313 Case 2: 2194, 2064	Case 1: 27 months Case 2: 15 weeks	Grand mal convulsions after accidental ingestion of clozapine overdose
[103]	USA	1 female, 1 male	38, 40	Caucasian	Schizophrenia/ not specified	olanzapine and paroxetine/not reported	Case 1: 2898 Case 2: 1500	Not reported	Drowsiness, minimal responsiveness, partial complex seizures with corresponding spike discharges on the EEG /CNS toxicity (sedation, weakness, dizziness, slurred speech, confusion)
[104]	USA	1 female	22	White	Schizophrenia	Haloperidol, olanzapine		58 days	Myoclonus resulting in unexpected falls and dropping things
[105]	Germany	1 male	30	Not reported	Schizophrenia	clomipramine	260	3 years	Obsessive Compulsive symptoms
[106]	Italy	1 female	29	Caucasian	Schizoaffective	Oral contraceptives	542	3 weeks	pericardial effusion and echocardiographic abnormalities (possible hypersensitivity reaction with eosinophilia, nausea, vomiting and palpitations)
[107]	Italy	1 male	27	Not reported	schizophrenia	lorazepam	425	12 days	Myocarditis presenting with fever, pharyngodynia, malaise, dyspnea. Also there were reported leukocytosis and slight cardiac enlargement on the chest X-ray.
[108]	United Kingdom	Male	41	Not reported	Paranoid schizophrenia	Lactulose, amitriptyline, sertraline, movicol when needed	Ante-mortem:560, post-mortem:3730	4 years	Constipation, aspiration of gastric contents and intestinal dilatation leading to death
[109]	USA	Female	61	Not reported	Chronic paranoid schizophrenia	Trazodone, fluoxetine, docusate sodium,	553	18 years	Paralytic ileus (nausea, vomiting, abdominal pain, distension)

						polyethylene glycol, bisacodyl suppository			
[110]	Italy	Male	45	Italian	Treatment-resistant residual schizophrenia	Lorazepam, sertraline	490	5 months	Intestinal occlusion (constipation, abdominal distention, biliary vomiting)
[111]	Netherlands	Male	44	Not reported	Schizophrenia	Lithium, omeprazole	1301	>10 years	Paralytic ileus (abdominal pain, nausea, vomiting, diarrhea
[112]	Netherlands	Male	46	Not reported	Chronic schizophrenia	Oxazepam, lansoprazole	2400	7 years	Extreme vomiting
	Netherlands	Male	37	Not reported	Chronic schizophrenia	Not mentioned	1056	4 years	Stomachache, diarrhea, incontinence
[113]	UK	Female	31	Caucasian	Schizoaffective disorder	Sodium valproate	CLOZ: 330<br>NCLOZ: 200	71 days	Neutropenia (reversed with lithium)
[90]	Germany	Female	70	Not reported	Delusional depression	Not reported	CLOZ: 285	6-8 weeks	Leukocytopenia.<br>CLOZ leukocyte levels (12.8) were 6-8 times higher than the mean of 10 controls with no history of leykocytopenia

Table S1B. Additional data from all included studies (cited in the main manuscript).

Reference	Mode of titration	Correlation of CLOZ dose with levels	Correlation of CLOZ dose with side effects	Use of specific scales/tools for the assessment of side effects
[34]	Titration by 25-50 mg increments every day or every other day up to assigned drug level	CLOZ dose and level significantly correlated (r=0.64, p<0.001). Dose correlated with EEG slowing (r=0.36, p=0.02)	Positive correlation with EEG slowing, but weaker than correlation with CLOZ levels	EEG recordings, Ordinal scale for sleepiness
[58]	Not precisely specified. Patients were switched from previous antipsychotic to CLOZ by stopping the first on the morning of one day and initiating CLOZ on the next. Mean final dose was 319.8±183.0mg/day, reached at 20.2±16.8 days, minus 1 week, during which CLOZ dosage should have been stable.	Not reported	No correlation	EEG recordings
[35]	Stable CLOZ dosage	Positive correlation between CLOZ dosage and CLOZ plasma levels (r=0.7, p<0.001)	No correlation	UKU side effects rating scale<br>ECG and EEG recordings<br>Pulse and systolic and diastolic BP measures
[39]	Titration over 12 weeks, increasing by 25-50mg every 1-2 days until reaching assigned range of serum levels	Positive correlation between CLOZ dosage and CLOZ levels (r=0.58, p<0.001)	Not reported	Extrapyramidal symptoms recorded on an anchored scale of seven items.<br>AIMS for the assessment of dyskinesias<br>Orthostatic hypotension, tachycardia, sleepiness, assessed on an ordinal scale (not validated).
[40]	Starting at 12.5 or 25 mg and increasing by no more than 25 mg every other day, over 6 weeks, with final dose: 75mg-800mg	No correlation between CLOZ dosage and CLOZ levels; positive correlation between CLOZ dosage and NCLOZ levels.	Not reported	No use of relevant scales; adverse effects rated by clinician as present vs absent
[41]	Stable CLOZ dosage	Close positive correlation between CLOZ dose (mg per kg) and CLOZ (r=0.60), NCLOZ (0.70), NOX (0.69) and total	No correlation	Systemic Assessment For Treatment-Emergent Events (SAFTEE),<br>AIMS, Barnes akathisia scale, Simpson-Angus extrapyramidal symptom scale

		metabolite and CLOZ levels (r=0.69), (p≤0.001)		
[42]	Titration over 3-4 weeks, reaching 300mg CLOZ daily. Further increase over another 4-5 weeks, reaching 500 mg CLOZ, in a subsample	Subjects on CLOZ dosage=300 mg/day had significantly lower plasma levels of CLOZ (p= 0.01) and NCLOZ (p=0.02)	Not reported	Laboratory, EEG and ECG measurements. Udvalg for Kliniske Undersogelser (UKU) Rating Scale
[75]	Stable CLOZ dosage	Not reported	Not reported	Liverpool University Neuroleptic Side Effect Rating Scale (LUNSERS)
[68]	Stable CLOZ dosage	Not reported	Not reported	Ray learning test, WAIS-III Digits test, , Card sorting test, , Phonetic Verbal Fluency Test
[43]	Not described	CLOZ dose (mg/kg) correlated with CLOZ (r=0.79, p=0.02) NCLOZ(r=0.82, p=0.01), and NOX (r=0.91, p=0.004) levels	Positive correlation with absolute and percent change in weight (r=0.7, p=0.03; r=0.9, p=0.005)	AIMS, Simpson-Angus EPS Scale, Subjective Treatment Emergent Symptoms Scale, Liver function tests, blood cell counts, EEG, ECG
[59]	Stable CLOZ dosage	Not reported	No correlation	CPS (Cognitive Performance Scale)
[62]	Titration over 16 weeks, starting at 25 mg on the first day, 50 mg on second and third days, 75 mg on the fourth and fifth days and 100 mg on each of the sixth and seventh days. Then titration upward by 100 mg per week for the first 3 weeks and thereafter by 200 mg per week until the assigned double-blind dose of 100mg, 300mg or 600mg	Not reported	Positive correlation at the end of the first 16-week trial (r=0.48, p=0.04), explaining only 25% of the variance of serum antimuscarinic activity Moderate positive correlation within-subject (r=0.69, p<0.001)	Checklist of clozapine side effects rated as present vs absent, focusing on those mainly related to antimuscarinic activity: constipation, hypersalivation, drowsiness/sedation, urinary disturbances. Measuring of serum antimuscarinic activity by the [^{3}H]QNB assay.
[60]	Stable CLOZ dosage	Not reported	No correlation	Y-BOCS, NIMH-G-OCS
[47]	Stable CLOZ dosage	Not reported	No correlation	EEG recordings
[44]	Not described	Positive correlation between CLOZ dose and plasma levels (p<0.01)	Not reported	Blood pressure, pulse and temperature mesurements
[64]	Initiating at 25-50 mg daily, increasing by 25mg 3 times a week, depending on efficacy and tolerance, until final dose of 468.3±179.3 over 8 weeks	Not reported	Inverse correlation with temperature between week 2 and week 7 of the study (r=-0.24, p<0.01)	Measurements of vital signs

[80]	CLOZ dosage not reported; possibly rapid titration, reaching CLOZ levels of 297±152 over 14±7 days, in patients who developed myocarditis at this time point	Not reported	Not reported	Echocardiography and relevant blood laboratory measures
[79]	Not reported; Measurements conducted at week 18 after initiation of CLOZ, at dosage 284.15±131.44	Not reported	Not reported	ECG recordings
[76]	Starting at 25 mg/day and increasing by 25mg/day once a week, over a period of 4 weeks, to final dose of 100 mg/day	Not applicable (all received the same CLOZ dose)	Not applicable	M-mode echocardiogeraphy measures and relevant blood laboratory measures
[72]	Stable CLOZ dosage for at least 8 weeks	Not reported	Not reported	HRV measures based on ECG recordings
[73]	Stable CLOZ dosage for at least a week	Not reported	Not reported	HRV measures based on ECG recordings
[48]	Stable dosage	Not reported	No correlation	Tachycardia recorded by 24- hour Holter ECG
[63]	Starting at 25 mg on the first day, 50 mg on second and third days, 75 mg on the fourth and fifth days and 100 mg on each of the sixth and seventh days. Then titration upward by 100 mg per week for the first 3 weeks and thereafter by 200 mg per week until the assigned double-blind dose of 100mg, 300mg or 600mg	Not reported	Positive correlation (p=0.048)	Weight measures
[81]	Stable dosage	Not reported	Not reported	CHOL, TG, HDL, LDL measurements
[65]	Patients on stable dose of clozapine for at least 2 months	Not reported	Negative correlation with AST plasma levels (r=-0.56, p=0.002)	Laboratory test measurements
[70]	Stable dosage	Not reported	Not reported	Lab measurements of insulin, IGF, IGFBP-1, blood glucose, triglycerides, cholesterol, HDL, LDL, leptin, IGF-I, IGFBP-1.
[49]	Stable dosage	Not reported	No correlation between dose and insulin levels	Glucose, insulin, IGF-I, IGFBP-1
[50]	Titration starting at 12.5 or 25 mg/d, escalating over 14 -21 days, depending on tolerance and response.	Not reported	No correlation	Weight measurement
[51]	Stable dosage	Not reported	No correlation with BMI	Measurements of BMI, lipid and fasting glucose blood levels. Evaluation of metabolic syndrome

[74]	Stable dosage	Not reported	Not reported	Measurement of weight, height, and blood tests for fasting glucose and lipid levels
[70]	Stable dosage	No correlation between CLOZ dose and CLOZ level	Not reported	Measurement of blood gucose lipid, insulin and C-peptide levels, calculation of HOMA-IR (Homeostasis Model Assessment Index for Insulin Reisistance
[71]	Titration in accordance with clinical efficacy and adverse effects, but not specifically described	Not reported	Not reported	UKU, Side Effect Rating Scale, ECG, urinanalysis, physical and neurologic examination, hematologic and biochemical blood tests
[77]	Stable dosage	Not reported	Not reported	Laboratory blood measurements
[66]	Starting at 25 mg on the first day, 50 mg on second and third days, 75 mg on the fourth and fifth days and 100 mg on each of the sixth and seventh days. Then titration upward by 100 mg per week for the first 3 weeks and thereafter by 200 mg per week until the assigned double-blind dose of 100mg, 300mg or 600mg	Not reported	Positive correlation between doses and prolactin levels in females only, but weaker than correlation with levels	Lab measurements of prolactin plasma levels
[46]	Initiation at 25 mg/d, increased by 25-50 mg every few days up to a median dosage of 300mg/d, over 2-3 weeks.	No correlation between weight-normalized CLOZ dosage and CLOZ or NCLOZ levels	Not reported	Medical evaluation and questioning of patients, relatives and nurses (present vs absent)
[52]	Not described	Not reported	No correlation	Lab liver function tests
[53]	Patients on stable dose, receiving clozapine for at least 3 months, therefore after titration	Not reported	No correlation	Measure of Colonic Transit Time (CTT) using radiopaque markers (ROM)
[54]	Stable dosage	Not reported	No correlation	Identification of laxative users by reviewing patient records, contacting GPs, interviewing 20% of the sample.
[36]	Stable dosage for at least 2 weeks before testing blood counts	Positive correlation between CLOZ dosage and CLOZ and NCLOZ levels (r=0.56, p<0.001)	No correlation	White cell and granulocyte counts
[37]	Initiation at 25 to 50 mg	Positive correlation between CLOZ dosage and blood	No correlation	Blood count measures

	daily in di vided doses given every 12 hours, and increase by 25 mg every 12 hours 3 times a week according to clinical indication	CLOZ(r=0.68) and NCLOZ(r=0.64) levels (p<0.001)		
[78]	Treatment at steady state	Not reported	Not reported	Blood laboratory test for blood cell counts
[38]	Titration over a period of 9 weeks, with final dose 75mg-600mg	Positive correlation between CLOZ dosage and CLOZ levels (r=0.34, p=0.00002), but not between CLOZ dosage and NCLOZ levels	No correlation	Leucocyte and neutrophil counts
[69]	Not described	Not reported	Not reported	Neutrophil counts
[67]	Stable dosage	Not reported	Positive correlation with neutrophil but not with leukocyte count	Blood laboratory tests
[55]	Not described	Not reported	No correlation	Measuring of anticardiolipin antibodies.
[57]	Stable dosage	Not reported	No correlation	UKU Side Effect Rating Sclae
[56]	Relatively stable dosage- only one increment ±50mg/d during the last 3 months	Not reported	No correlation	Abnormal Involuntary Movements Scale (AIMS), RDC severity criteria for tardive dyskinesia, Extrapyramidal Side Effects rating scale (EPSE), Barnes Akathisia rating scale (BARS), Antipsychotic Non-Neurological Side Effects Rating Scale (ANNSERS)

Table S2. General scores of adverse effect scales or combinations of adverse effects in relation to clozapine plasma levels.

Reference	Type of study	Location	Patients (Males/ Females)	Age (Mean) (years)	Race	Diagnosis	Comedication	Averaged serum levels of clozapine (ng/ml)	Duration of CLOZ exposure	Reported Side effects	Correlation to CLOZ plasma levels	Jadad score
[56]	Cross-sectional	UK	103 (71/32)	39.3±8.8	White: 60 (58%)	Schizophrenia (n=101), Schizoaffective (n=2)	Mood stabilizer (31%), Anticholinergic (18%), antidepressant (16%) other antipsychotic (5%), anxiolytic or hypnotic (5%)	CLOZ: 530 ±370, NCLOZ: 310 ±190	Median (range): 30 (3-156) months	Parkinsonism (18%), Akathisia (4%), Tardive Dyskinesia (5%), Non neurological side effects including cardiovascular, gastrointestinal, sexual genitourinary and others (moderate/severe, ≥1: 77%of patients)	Positive but weak correlation between CLOZ levels and total ANSSERS score (Pearson correlation=0.29, p<0.004), and between CLOZ levels and number of moderate and severe side effects (Pearson correlation=0.23, p<0.03) Patients with CLOZ levels over 250ng/ml were more likely to have ≥1 moderate or severe side effects than those with concentrations below this level (63/76 vs 12/23, p<0.01)	1
[43]	Open label	USA	6 (2/ 4)	13.3±2.7 (range 9-16)	Caucasians (n=2) Afro-Americans (n=2), Hispanic (n=1), Pacific Islander (n=1)	Childhood onset schizophrenia	No concomitant medications	Crude (ng/mL): 289 ± 116, Normalized (ng/mL-mg-Kg): 99 ± 37.3	6 weeks	Adverse effects included sedation (1/6), enuresis (1/6), tachycardia (4/6), sialorrhea (5/6), reduced neutrophil count (1/6), increased hepatic transaminases (1/6).	Total number of moderate/severe side effects correlated positively with CLOZ+NCLOZ (r=0.4, p=0.002), NCLOZ (r=0.6, p=0.002), and CLOZ+NCLOZ+NOX levels (r=0.4, p=0.03)	0

										Number of side effects: 3.8±0.75, Number of moderate/severe side effects: 2.5±1.0		
[46]	Prospective, open follow-up	Italy	45 completed the study (35/10)	19-65	Not specified	Chronic schizophrenia	No Comedication allowed	CLOZ: 385±183 (range 147-974) NCLOZ: 174±84 (range: 43-445)	12 weeks	Hypersalivation (n=3), constipation (n=3), tachycardia (n=2), dizziness (n=1), sedation (n=4), weight gain (n=3)	No	1
[41]	Cross-sectional	USA	44 (33/ 11)	36.6± 9.1 (range 20-54)	Not specified	Schizophrenia (43%), schizoaffective bipolar (32%), bipolar (14%), schizoaffective depressed (7%), major depression-psychotic (4%),	benzodiazepines, lithium, antidepressants,	CLOZ: mean=297 (median: 291), among 68 samples. Subsample not exposed to fluoxetine or valproate (n=27): 239±159	2.15± 2.30 years	SAFTEE scale ADRs	No	0

Table S3. Nervous system and psychiatric adverse effects in relation to CLOZ plasma levels.

Reference	Type of study	Location	Patients (Males/Females)	Age (Mean) (years)	Race	Diagnosis	Comedication	Averaged serum levels of clozapine (ng/ml)	Duration	Reported Side effects	Correlation to clozapine plasma levels	Jadad score
**Neurologic and Psychiatric Adverse Effects**												
[34]	prospective randomized	USA	50 (39/11)	38 (range: 21-56)	white(n=26) black(n=24)	chronic schizophrenia or schizoaffective disorder	No sedative medications permitted (barbiturates, benzodiazepines) Rarely given doses of haloperidol or fluphenazine during the first 2 weeks of clozapine titration.	group I (n=16): 50-150 group II (n=22): 200-300, group III (n=12): 350-450	12 weeks	Seizures: 3/50 (6%) (2 patients with preexisting history of seizures) EEG abnormalities, Overall: 24/45 (53%), More severe than borderline: 15/45 (33%). Spike/sharp activity Slowing Sleepiness	EEG abnormalities, more severe than borderline, rate: group III: 73%vs Group I: (20%) and Group II: 21% (p=0.006) Severity: group I 0.9±1.8, group II 1.0±1.5, group III 3.4±1.9 (p<0.001) Spike/sharp activity: no correlation Slowing: more slowing in group III vs group II and I (p=0.049), positive correlation with levels (r=0.44, p=0.002), possible cutoff: 300ng/ml Sleepiness: positive correlation with levels (r=0.33, p=0.029). The EEG slowing correlated with observed sleepiness.	2
[58]	Prospective, observational	Austria	29 (18/ 11)	31.7±10.2	Not specified	Schizophrenia (n=22) Schizoaffective (n=7)	No psychotropic /anticholinergic comedication allowed	Whole Sample(n=29): 161.3±150.0 Group 1 (n=14): 81.6±64.6, Group 2 (n=15): 235.7±169.8	20.2±16.8 days	EEG alterations (53%), with categorization of severity. Severe changes with intermittent spark transients: 27.6%. No seizures observed.	Plasma levels significantly different among groups according to severity of EEG changes. Group 1(n=14): degree 0-1 , plasma levels: 81.6±64.6 ng/ml (95%CI=44.3-118.9). Group 2 (n=15): degree 2-4, plasma levels: 235.7±169.8 mg.ml (95% CI: 141.7-329.7) (p=0.0009)	0
[35]	Cross-sectional, blinded for	Denmark	30 (21/ 9)	37,6 ± 1,67	Not specified	Schizophrenia	No other neuropleptic allowed except levomepromazine or	Median CLOZ: 351 (231-615) (range: 64-1824)	2.5 (1.0-9.0) years	EEG changes	Severity correlated to plasma CLOZ (r = 0.43; P < 0.05) but not NCLOZ levels.	0

	EEG measures						chlorprothixene for sedation up to 100mg/day. Other medication as usual				CLOZ concentrations ≥ 1306 ng/ml lead to progressive gradual EEG changes	
[39]	Prospective, randomized, double-blind	USA	56 (41/15)	3 (range 21-56)	Not specified	Schizophrenia	All psychoactive medication tapered off, except for haloperidol and phluphenazine and an antiparkinsonian agent. Two patients with a history of seizures stayed on valproate throughout the study.	low: 91±15 (50-150), medium: 251±13 (200-300), high: 396±16 (350-450)	12 weeks	Sleepiness (score>1) in 38% and 30% on weeks 6 and 12 respectively. EPS	Trend of sleepiness and serum level to correlate at week 6 (p=0.08), but no significance at week 12. EPS improved overtime, with no group-by-time interactions.	2
[40]	Prospective, longitudinal, observational, partly double-blind(n=22) partly open label (n=32)	USA	54 (34/ 20)	Range: 8-18	white (n=25) African American (n=17), Hispanic (n=4), Asian (n=2), other (n=6)	Childhood-onset schizophrenia	Medication washout and medication free period of 1-3 weeks prior treatment	Week 6: clz 455±285.1, nor c+lz 302.4±142.2	6 week treatment at first and then 2-6 years follow-up	EEG abnormalities on week 6: Slowing 11%, epilepitiform 11%, seizures: 6%. Akathisia: 15%.	Rates of side effects were not directly associated with CLZ or nor CLZ blood levels or their ratio.	0
[41]	cross-sectional	USA	44 (33/ 11)	36.6± 9.1 (range 20-54)	Not specified	Schizophrenia (43%), Schizoaffective bipolar (32%), Bipolar (14%), Schizoaffective depressed (7%), Major depression-psychotic (4%),	Medicines allowed:benzodiazepines, lithium, antidepressants, other medically indicated agents, except drugs known to alter clearance of neuroleptics, i.e phenytoin and cimetidine	CLOZ: mean=297 (median: 291), among 68 samples. Subsample not exposed to fluoxetine or valproate (n=27): 239±159	2.15± 2.30 years	Sedation (presence vs absence)	No	0
[42]	Prospective, non randomized, double blind, observational	Hong-Kong	51 (38/ 13)	37.61 ± 8.68 (range 21-63)	Not specified	Schizophrenia (n=48) and schizoaffective (n=3),	No psychotropics or anticholinergics allowed. Chloral hydrate allowed for sedation Lorazepam needed short term in two patients Valproate was given to patients with epileptiform activity in EEG, myoclonus or	Week 6: Clozapine: 470.20±234.2,range 100-1220 Norclozapine:233.06±105.56, range 70-670, Week 12: cloz 681± 390.71, range 220-1920, NCLOZ: 297.8 ± 146.49 range 8-720	12 weeks	EEG abnormalities, BARS, AIMS, SAS scores(akathisia, extrapyramidal symptoms)	No significant correlation between plasma levels and EEG abnormalities on week 6, BARS, AIMS, SAS scores on weeks 6 and 12, Negative correlation with sedation at week 6, which was clinically implausible	0

							seizure (number of patients not specified)					
[47]	Retrospective, observational	Korea	71 (43/28)	37.7±11.7	Not reported	Schizophrenia (n=59)<br>Bipolar disorder (n=12)	Valproate (n=25, 35.2%)<br>Mood stabilizers (lamotrigine, valproate, lithium, topiramate) (n=31)<br>Antipsychotics, antidepressants, benzodiazepines, or mood stabilizers in combination (n=68)	Clozapine: 429.4±264.1<br>Norclozapine: 197.8±132.6	4.6±4.9 years	EEG abnormalities:<br>Slow waves (focal slowing, theta waves)<br>Spikes<br>Patients with EEG abnormalities: 51 (71.8%, focal slowing: 39,2%, theta waves: 49.0%, spikes: 16.9%)	Positive correlation with CLOZ levels (p0.008). No correlation with NCLOZ levels (p=0.12)<br>Patients with CLOZ levels>600ng/ml had higher rate of EEG abnormalities (93.8%) than those with levels<600ng/ml (65.5%) (p=0.02)	0
[75]	cross-sectional	Finland	237 (136/ 101) (blood sample available in 190)	Age: 42.5(range : 20-65)	Caukasian (Finnish)	Schizophrenia (F20), schizophreniform (F23.1-2), schizoaffective (F25), delusional disorder (F22)	Clozapine monotherapy: 65.4%,<br>Cloz+atypical: 22.5%,<br>Cloz+typical: 9.7%,<br>cloz+typical+ atypical: 1.7%	Men: 722±366, Women: 886±480 (p=0.03) Total sample: 778±444.57	3-12 months (1.7%),<br>1-5 years (32.5%),<br>5 years (57.8%),<br>Not specified (8%)	The Depression/anxiety score, comprising difficulty in concetrating, tension, difficulty remembering things, depression, restlessness, difficulty getting to sleep, was higher in patients with clozapine+norclozapine concentration of at least 2.2 μmol/l (719ng/ml) (n=92) compared with patients with lower serum concentrations (n=95) (9.7 ±4.8 points vs 7.5± 5.1, P=0.004). No significant correlation of other side effects (sedation factor, dreaming item, lack of emotion item, sympathichotonia factor) with serum concentrations	Yes, only with the Depression/Anxiety score	0

[68]	Single-blind, cross-sectional	Spain	19 (11/ 8)	Group I: 45 ±10.3, Group II 47.2 ± 7.5	Not specified	Schizophrenic Disorder or Schizoaffective disorder	No psychotropic comedication	Group I: Clz ≥300 Group II: Clz <300	≥ 5 years	cognitive performance	No relationship between clozapine plasma levels and cognitive performance. Tendency to significance regarding the executive test (31% of variability of number of attempts in the WCST was explained by clozapine plasma levels)	0
[43]	Open label	USA	6 (2/ 4)	13.3±2.7 (range 9-16)	Caucasians (n=2) AfroAmericans (n=2), Hispanic (n=1), Pacific Islander (n=1)	Childhood onset schizophrenia	No concomitant medications	Crude (ng/mL): 289 ± 116, Normalized (ng/mL-mg-Kg): 99 ± 37.3	6 weeks	Adverse effects included sedation (1/6), among others.	no	0
[59]	Retrospective analysis of clinically collected cross-sectional data.	Canada	73 (48/ 25)	41.6 ± 12.0	Not specified	Schizophrenia (n= 52, 71.2%) Schizoaffective disorder (n=11, 15.1%), Psychotic disorder not otherwise specified (n= 9, 12.3%) Delusional disorder (n= 1, 1.4%)	Not reported	All subjects (n=73): 458.5 ± 248.8 Low cognitive impairment (n=57): 437.1 ± 249.6 High cognitive impairment (n=16): 534.7 ± 237.7	≥3 months	cognitive impairment	Sixteen subjects (21.9%) had high cognitive impairment and the rest had low cognitive impairment. Age and clozapine levels were associated with high cognitive impairment, as well as clozapine/desmethylclozapine raitio (OR: 7.3).Yes	0
[56]	Cross-sectional	UK	103 (71/32)	39.3±8.8	White: 60 (58%)	Schizphrenia (n=101), Schizoaffective (n=2)	Mood stabilizer (31%), Anticholinergic (18%), antidepressant (16%)other antipsychotic (5%), anxiolytic or hypnotic (5%)	CLOZ: 530 ±370, NCLOZ: 310 ±190	Median (range): 30 (3-156) months	Memory and concentration problems (38%), Nighttime sleep problems (32%) Parkinsonism (18%), Akathisia (4%), Tardive Dyskinesia (5%),	No correlation	1
[62]	Double-blind, prospective.	USA	40 (39 completers), (sex not reported)	not reported	Not specified	Schizophrenia and schizoaffective disorder	After a 4 week Haloperidol trial and 1 week wash out,	End of first trial (16 weeks): 335±340	First trial:16 weeks, second	serum antimuscarinic activity, drowsiness/sedation (present vs absent)	Clozapine levels were very good predictors of serum antimuscarinic activity in doses of 300 mg/d or higher.	2

	Randomized to clozapine doses						randomized at 100, 300, 600mg/day of clozapine		trial:16 weeks		Sedation showed no significant association with serum antimuscarinic activity.No	
[60]	Retrospective, naturalistic, 1-year study/cross-sectional	Taiwan	102 (59/43)	37.9 ± 9.3	not specified	Schizophrenia	Fluvoxamine (n=8), fluoxetine (n=2), sertraline (n=2), paroxetine (n=1), valproate (n=15)	Patients with OCS: 595.1±364.9 (range 84-1491) Patients without OCS: 433.5±252.8 (range 82-1273)	Patients with OCS: 81.8 ± 32.2 months, Patients without OCS: 56.1 ± 40.6 months	Thirty-nine patients (38.2%) presented with OCS, and, of these, 29 patients (28.4%) were classified as clozapine-induced, with an average latent period of 39.8 ± 22.5 months.	plasma concentration of clozapine was significantly higher in patients with OCS than in those without (P=0.001)	0

Table S4. Cardiovascular adverse effects in relation to CLOZAPINE blood levels.

Reference	Type of study	Location	Patients (Males/Females)	Age (Mean)	Race	Diagnosis	Comedication	Averaged serum levels of clozapine (ng/ml)	Duration	Reported Side effects	Correlation to clozapine plasma level	Jadad score
[35]	Cross-sectional	Denmark	30 (21/ 9)	37,6 ± 1,67	Not specified	Schizophrenia	No other neuropleptic allowed except levomepromazine or chlorprothixene for sedation up to 100mg/day. Other medication as usual	Median S-Clozapinea: 351 (231-615) (range: 64-1824)	2.5 (1.0-9.0) years	Increased pulse rate(>80bpm): 73%, Tachycardia (>100bpm): 23% Orthostatic hypotension: 17%	No	0
[39]	Prospective, randomized, double-blind	USA	56 (41/15)	38 (range 21-56)	Not specified	Schizophrenia	All psychoactive medication tapered off, except for haloperidol and phluphenazine and an antiparkinsonian agent. Two patients with a history of seizures stayed on valproate throughout the study.	low: 91±15 (50-150), medium: 251±13 (200-300), high: 396±16 (350-450)	12 weeks	Tachycardia: 48%(n=27) Orthostaic hypotension: 5%(n=3) No significant relationships between plasma levels and tachycardia or orthostatic hypotension	No	2
[40]	Prospective, longitudinal, observational, partly double-blind(n=22) partly open label (n=32)	USA	54 (34/ 20)	Range: 8-18	25 white, 17 African American, 4 Hispanic, 2 Asian 6 other	Childhood-onset schizophrenia	Medication washout and medication free period of 1-3 weeks prior treatment	Week 6: clz 455±285.1, nor clz 302.4±142.2	6 week treatment at first and then 2-6 years follow-up	Hypertension (>140/90mmHg): 6%, Orthostatic hypotension: 7%, Tachycardia (>120bpm): 28%	No	0
[42]	Prospective, non randomized, double blind, observational	Hong-Kong	51 (38/ 13)	37.61 ± 8.68 (range 21-63)	Unspecified	Schizophrenia (n=48) and schizoaffective (n=3),	No psychotropics or anticholinergics allowed. Chloral hydrate allowed for sedation Lorazepam needed short term in two patients Valproate was given to patients with epileptiform activity	Week 6: Clozapineapine:470.20±234.23, range 100-1220 Norclozapine:233.06±105.56, range 70-670, Week 12: clozapine 681± 390.71 ng/mL (range 220-1920 ng/mL), norclz 297.8 ± 146.49 ng/mL (range 8-720 ng/mL)	12 weeks	QTc alterations	no	0

							in EEG, myoclonus or seizure (number of patients not specified)					
[44]	Prospective, Open label	Germany	32 (17/ 15) (plasma levels available in only 5)	Not reported	Not specified	Paranoid-hallucinatory schizophrenia (n=12) schizoaffective (n=8) manic (n=3) schizophreniform (n=9)	Not reported	200-300 ng/ml (divided into three groups I: <30 ng/ml, II: 30-100 ng/ml, III: >100 ng/ml for paranoid-hallucinatory schizophrenic patients and manic syndrome)	30 days	In all of the patients decreases in systolic blood pressure, diastolic blood pressure and blood pressure amplitude occurred in the standing position and simultaneously pulse rate was elevated, most pronounced on day 3. After 10 and 20 days of treatment an adaptation occurred. There existed a positive relationship between plasma levels and severity of orthostatic dysregulation at day 6 and 10. A slight mean increase in body temperature was measured during treatment with clozapine	Yes, with severity of orthostatic dys-regulation	0
[43]	Open label	USA	6 (2/ 4)	13.3±2.7 (range 9-16)	Caucasians (n=2) AfroAmericans (n=2), Hispanic (n=1), Pacific Islander (n=1)	Childhood onset schizophrenia	No concomitant medications	Crude (ng/mL): 289 ± 116, Normalized (ng/mL-mg-Kg): 99 ± 37.3	6 weeks	Adverse effects included tachycardia (n=4)	no	0
[64]	Prospective longitudinal	Canada	37 (26/ 11) (30 completers)	Not reported	not specified	Schizophrenia	Only lorazepam allowed, at 2mg daily for agitation and agression	Mean serum clozapine level by week (1-8): 63.5±46.0 199.3±149.1 251.9±178.0 300.7±200.2 316.2±189.4 364.7±195.6 351.9±176.2 379.5±156.5	First 8 weeks of administration	Blood pressure, temperature, pulse	The blood pressure and pulse did not change significantly from baseline to week 8.	0

										Temperature was inversely related to clozapine dose (p<0.003). Higher nor-clozapine to clozapine ratios were associated with higher BP measures (p=0.002). The magnitude of these relationships is weak (r<0.30). There is a tendency to autonomic dysregulation during clozapine use.	
[80]	Cohort, prospective study, open	Australia	503 (397/ 106)	44±12	not specified	Schizophrenia	Not reported	475±236	Mean follow-up of 9±6 years	Myocarditis: 3%(n=14), at 14±7 days, with mean clozapine level 297±152. Sudden death: 2%(n=10), at 5±4 years, mean clozapine level 439±198.	No	0
[79]	Retrospective, review of case records	Spain	82 (58/24)	31.21 ±9.59	not specified	schizophrenia (n=65), schizoaffective (n=10), Psychotic disorder NOS (N=2), Bipolar (N=4), Major Depression n=1).	None: 52.44% Antidepressants: 19.51% Mood stabilizers:31.71% Stimulants: 1.22%	CLZ: 305.56±299.64, nor CLZ: 160.23±105.12	18 weeks	No significant interaction between clozapine levels>400ng/ml and QTc plrolongation. No differences detected in the prevalence of prolonged QTc or in mean QTc before and after clozapine treatment.	No	1

[76]	Preliminary prospective study	Italy	20 initially, 15 completers (5 discontinued due to siallorhea, nausea, or hypotension) (8 /7)	35.5±11.0	not specified	Schizophrenia	One other antipsychotic, one mood-stabilizing drug or a benzodiazepine	CLZ: 124±70.8 nor CLZ: 52.3±35.7	4 weeks	Heart Rate increased significantly by an average of 10.4% (p=0.013), 20% of cases had > 100/min PAP was >33mmHg in 13.3% of cases A-wave velocity exceeded 77cm/sec in 26.7% of cases, Myocardial Performance Index (MPI) exceeded 0.44 in 100% of cases. E/A ratio was normal (100%) Overall findings consistent with lees efficient LV functioning, in a large poportion of patients with low to moderate clozapine serum levels. No dose-response relationship mentioned	No	0
[72]	Cross-sectional	Germany	80 Clozapineapine: 40 (20/20) Healthy controls: 40 (20/20)	Patients: 40.7 (range:21-68) Controls: 41.2 (range: 20-64)	Not reported	Schizophrenia Schizoaffective disorder	none	290	>8 weeks	HRV	Yes, negative (inverse) correlation	0
[73]	retrospective	Germany	33 (19/14) (Total sample: 65)	42.1 (range: 19-73)	Not reported	Schizophrenia, schizoaffective disorder	Haloperidol, benzodiazepines, SSRIs, Carbamazepine	331±294 (range:65-1475)	>1 week	HRV	Yes, negative (inverse) correlation	0
[48]	Cross-sectional	Sweden	30 (20/10)	33.5 (range: 26-41)	Not reported	Not reported	Not reported	451 (range: 337-569) (n=29)	7 (range:3-13)	Persistent Tachycardia	No	0

Table S5. Metabolic and endocrine adverse effects in relation to clozapine plasma levels.

Reference	Type of study	Location	Patients (Male/ Female)	Age (Mean) (Years)	Race	Diagnosis	Comedication	Averaged serum levels of clozapine (ng/ml)	Duration	Reported side effects	Correlation to clozapine plasma levels	Jadad score
[63]	Prospective double-blind randomized	USA	50 (22/28)	44.8±9.6	Caucasian (n=43) African American (n=7)	Schizophrenia (n=39) Schizoaffective (n=11)	None	CLOZAPINE:> 350 in responders	29.7 ± 13.2 weeks(16 weeks, 32 weeks, 48 weeks, based on response status)	Weight gain (mean=1.6 kg)	Non significant for CLOS and NCLOZAPINE levels. In nonsmokers (n = 8) and after controlling for baseline BMI, sex, and clozapine levels, the positive partial correlation between weight gain and NCLOZAPINE levels, r = 0.89 (P = 0.046).	
[81]	Prospective observational open-label	Ireland, UK	49 (33/16)	37.4 ±9.3, (range 22–57)	Not specified	Schizophrenia	Not reported	500±280 (range 70–1360).	32.6 ± 6.6 weeks (23-62 weeks)	Weight gain, waist circumference, fasting total serum cholesterol, serum TG, LDL cholesterol and HDL cholesterol (correlation testing reported only for serum TG increase)	No correlations were found between change in serum TG levels and serum clozapine levels (r=−0.04, p=0.77) at follow-up	0
[65]	Cross-sectional	Australia, Singapore	40 (18/22)	38.2 ± 11.3 (range 22-74)); Caucasians:40.2±8.6, Asians:36.3± 13.4	Caucasians [n=20, (16/4)] Asians [n=20 (2/18)]	Schizophrenia	Not reported	Caucasians: 415.3 ±185.8 Asians: 417.1±290.8	≥6 months	Lipid profiles, fasting glucose levels	No correlation found between CLOZAPINE levels and total cholesterol, LDL, triglyceride or serum glucose levels	0
[70]	Cross-sectional	Sweden	34 (13/5)	46 (29-63)	Not specified	Schizophrenia, schizoaffective disorder	Not reported	Median: 359.1 (range 60.5–810.46)	At least 6 months Median: 5.3 years (range 0.5–16.3 years)	Fasting blood samples for insulin, C-peptide, insulin-like growth factor I, insulin-like growth factor binding protein-1, leptin, glucose and lipids	Positive correlation between CLOZAPINE levels and levels of insulin, C-peptide (r=0.51, P=0.03 and r=0.48, P=0.04, respectively), and triglycerides ((rs=0.50, P=0.03), but not with NCLOZAPINE levels.. No correlation between CLOZAPINE or NCLOZAPINE levels with	0

											IGF-I, IGFBP-1, leptin, glucose, cholesterol, HDL, LDL.	
[49]	Cross-sectional	Sweden	41 (7/6)	35 (26-47)	Not mentioned	Schizophrenia, schizoaffective disorder, delusional disorder	Comparison between clozapine and classical antipsychotics (perphenazine or zuclopenthixol)	28.8-721	2.7 (range 0.5-7.3 years)	Fasting blood samples for glucose, insulin, growth hormone (GH)-dependent insulin-like growth factor I (IGF-I), and insulin-dependent insulin-like growth factor binding protein-1	insulin levels positively correlated to the serum CLOZAPINE concentration	0
[50]	Open, prospective	USA	74 (46/28). Plasma clozapine levels determined in a subsample (n=26)	Males: 34.7±8.1<br>Females:36.2±11.6	Not specified	Schizophrenia	Benztropine (n=6), diphenylhydantoin (n=2), fluoxetine (n=1), divalproate (n=1)	6 weeks: 388±242<br>6 months: 444±355	6 months	Weight gain	No	0
[51]	Cross-sectional	Canada	60 (47/13)	36.5±11.3	Caucasian (93%)<br>Asian (n=2),<br>Fiirst Nations (n=2)	Schizophrenia (n=46), schizoaffective (n=13), delusional disorder(n=1)	Antipsychotics (61.9%), Antidepressants (14.3%), Mood stabilizers (21.4%)	CLOZAPINE::1613.57± 976.05<br>NCLOZAPINE:964.60±976.05	Stable clozapine therapy for at least 6 months	Metabolic syndrome<br>BMI (obese/overweight)	No correlation with bmi/ Positive correlation with metabolic syndrome. 11% increase in the odds per 100ng/ml, levels 1.5 times higher in those with vs without metabolic syndrome	0
[74]	retrospective	UK	100 (59/41)	Males: 36.9 (95% CI: 33.9-39.8)<br>Females: 39 (95% CI: 35.4-42.7)	Not reported	Schizophrenia (n=82)<br>Schizoaffective disorder (n=18)	Aripiprazole (n=6)<br>Amisulpride (n=4)<br>Haloperidol (n=2)<br>Of the females: oestrogen containing contraceptive pill (n=5), oestogen containing hormone replacement treatment	CLOZAPINE:<br>Males: 440 (10th-90th percentile: 260-700)<br>Females: 490 (10th-90th percentile:270-790)<br>NCLOZAPINE:<br>Males: 310 (10th-90th percentile:260-350)<br>Females: 310 (10th-90th percentile:270-340	Males: 4.4 (95% CI: 1.2-10.3) years<br>Females: 5.1 (95% CI: 2.3-7.9) years	BMI<br>Fasting blood glucose<br>HDL	Yes, positive with BMI and fasting blood glucose<br>Not with HDL	0
[45]	Cross-sectional	Sweden	17 (12/5)	Median:41 (range: 29-36)	Caucasian	Schizophrenia (n=16)<br>Schizoaffective(n=1)	Benzodiazepines (n=4), and/or levomepromazine (n=3) and/or lithium (n=1)	CLOZAPINE: 392(69-918)<br>NCLOZAPINE: 288 (88-641)	6.9 years (range: 0.7-16.3 years)	Elevated blood glucose (n=2), elevated levels of insulin (n=10),	Positive correlation between CLOZAPINE levels and insulin (r=0.53, p=0.03), C-peptide (r=0.51, p=0.04), and	0

										elevated levels of C-peptide (n=13), elevated triglycerides (n=8), and cholesterol (n=6). Also, homeostasis model assessment index for insulin resistance (HOMA-IR)	triglyceride levels (r=0.46, p=0.06, trend). No correlation with cholesterol. No correlation between NCLOZAPINE levels and hormone or lipid levels.	
[71]	Prospective, randomized	Taiwan	68 (20/48) Coadministration group:34 (10/24) Monotherapy group: 34(10/24)	Coadministration group: 32.9±8.5 Monotherapy group: 35.1±9.4	Han Chinese	Schizophrenia	Medication interfering with body weight, lipid or glucose metabolism or clozapine disposition were not allowed (ie. Lithium, propranolol, carbamazepine, valproate, tricyclic antidepressants, other SSRIs)	Coadministration group: CLOZAPINE: 509.8±281.1 NCLOZAPINE:179.0±95.8 Monotherapy group: CLOZAPINE: 502.0±220.6 NCLOZAPINE: 242.8±100.3	12 weeks	Serum glucose, cholesterol, and TRG levels Weight gain	No correlation with CLOZAPINE levels. Significant correlation of weight gain (r=0.27, p=0.026), blood sugar (r=0.34, p=0.005) and TRG levels(r=0.27, p=0.028) with NCLOZAPINE levels. Tendency to correlation between cholesterol levels and NCLOZAPINE levels (r=0.21, p=0.07, trend)	2
[77]	Cross-sectional, controlled	Turkey	70 Patients: 38 (17/21) Controls: (14/18)	Patients:40.94±10.15 Controls: 40.09±1.67	Not reported	Schizophrenia or schizoaffective	Not reported	CLOZAPINE: 594.90±492.90 NCLOZAPINE: 220.33±182.55	At least 4 months	Laboratory blood measures	No correlation with GLU, TG, HDL, LDL, Positive correlation of CLOZAPINE levels with TC (r=0.335, p=0.040)	0
[41]	cross-sectional	USA	44 (33/ 11)	36.6± 9.1 (range 20-54)	Not specified	schizophrenia (43%), schizoaffective bipolar (32%), bipolar (14%), schizoaffective depressed (7%), major depression-psychotic (4%),	Medicines allowed:benzodiazepines, lithium, antidepressants, other medically indicated agents, except drugs known to alter clearance of neuroleptics, i.e phenytoin and cimetidine	CLOZAPINE: mean=297 (median: 291), among 68 samples. Subsample not exposed to fluoxetine or valproate (n=27): 239±159	2.15± 2.30 years	Excess weight	No	0
**Endocrine system**												
[66]	Double-blind	USA	35/40 (16/19)	49 females (32-60 years old), 42	90% (17/19) of females	Schizophrenia, schizoaffective disorder	Comparison between haloperidol and	400-1600	16-week	Hyperprolactinemia	For every 100 ng/ml increase in plasma clozapine levels, average increments	2

	dose-response			males (31-58 years old)	were Caucasian and 10% (2/19) of females were African-American, 81%t (13/16) of males were Caucasian and 19 % (3/16) of males were African-American		clozapine (4 subjects were not treated with Haloperidol for 4-weeks due to history of intolerance to haloperidol so they continued under their baseline typical antipsychotic)				in prolactin levels of 0.45 ng/ml in females and of 0.15 ng/ml in males were recorded	
[75]	Cross-sectional	Finland	237 (136/101) CLOZAPINE levels measured in 190 (112/78)	42.5 (20-65)	Native Finnish	Schizophrenia, schizophreniform, schizoaffective disorder, delusional disorder	Clozapineapine Monotherapy: 65.4%, Clozapine+atypical: 22.5%, Clozapine+typical: 9.7%, Clozapine+typical + atypical: 1.7%	Men (722±366) and women (886±480)	3-12 months (1.7%), 1-5 years (32.5%), .5 years (57.8%), unspecified (8%)	Menstrual problems	No correlation with CLOZAPINE+NCLOZAPINE concentration	1
[77]	Cross-sectional, controlled	Turkey	70 Patients: 38 (17/21) Controls: (14/18)	Patients:40.94±10.15 Controls: 40.09±1.67	Not reported	Schizophrenia or schizoaffective	Not reported	594.90±492.90 220.33±182.55	At least 4 months	Laboratory blood measures	No correlation with TSH, FT4, PRL. Negative correlation with FT3 (r=-0.373, p=0.021).	0

Table S6. Gastrointestinal adverse effects in relation to CLOZAPINE blood levels.

Reference	Type of study	Location	Patients (Males/ Females)	Age (Mean) (Years)	Race	Diagnosis	Comedication	Averaged serum levels of clozapine (ng/ml)	Duration	Reported Side effects	Correlation to clozapine plasma levels	Jadad score
[41]	Cross-sectional	USA	44 (33/11)	36.6± 9.1 (range 20-54)	Not specified	Schizophrenia (43%), schizoaffective (32%) bipolar (14%), major depression-psychotic (7%)	Medicines Allowed: benzodiazepines, lithium, Antidepressants, other Medically Indicated agents, Except drugs Known to alter Clearance of Neuroleptics, i.e Phenytoin and Cimetidine	Median 291, range: 15-726	2.15± 2.30 years	Nocturnal sialorrhea, drooling, parotid swelling Constipation	No	0
[46]	Prospective, obsevetional follow-up	Italy	45 completed the study (35/10)	19-65	Not specified	Chronic schizophrenia	No Comedication allowed except for benzodiazepines Occasionally	CLOZAPINE: 385±183 (range 147-974) NCLOZAPINE: 174±84 (range: 43-445)	12 weeks	Hypersalivation, constipation	No	1
[52]	Prospective	Austria	238 (153/85) Patients on CLOZAPINE: 167 (101/66)	CLOZAPINE subsample: 31.37±11.8	Not specified	Not reported	Not reported	165.4±163.4	18 weeks	Pathologic liver function tests (LFTs): SGOT, SGPT, GGT, ALP, Bilirubin	Yes, for SGPT only.	0
[35]	Cross-sectional, naturalistic	Aarhus, Denmark	30 (21/9)	Range 22-55	Not mentioned	Schizophrenia (schizoaffective:5, hebephrenic:5,paranoid: 8, latent:1, catatonic:2, simplex:2, unspecified:7)	Nortriptyline:1, levomepromazine:5, clonazepam:6, hyoscyamine:4, oxazepam:3, chlorprothixene:1, phenobarbital:1, nitrazepam:1, biperiden:2, orphenadrine:1, benztropine:2, diazepam:2, piroxicam:1, disulfiram:1	Median: 1076 (range 706-1882) NCLOZAPINE/CLOZAPINE ratio: 0.77±0.17	Median: 2.5 (range 1.0-9.0) years	Increased liver enzyme activity (increased GGT, ALP, AST, ALT)	No	0
[77]	Cross-sectional, controlled	Turkey	70 Patients: 38 (17/21) Controls: (14/18)	Patients:40.94±10.15 Controls: 40.09±1.67	Not reported	Schizophrenia or schizoaffective disorder	Not reported	594.90±492.90 220.33±182.55	At least 4 months	AST, ALT	No	

[65]	Cross-sectional	Australia, Singapore	40 (18/22) ; 20 Caucasians (16/4), 20 Asians (2/18)	38.2 ± 11.3 (range 22-74); Caucasians:40.2±8.6, Asians:36.3± 13.4	Caucasians [n=20, (16/4)] Asians [n=20 (2/18)]	schizophrenia	Not reported	Caucasians:415.3±185.8, Asians:417.1±290.8	≥6 months	Elevated levels of alanine (ALT) and aspartate (AST) transferases	No	0
[43]	Open-label trial	USA	6 (2/4)	13.3±2.7 (range 9-16)	Caucasian:2, African American:2, Hispanic:1, Pacific Islander:1	Childhood-onset schizophrenia	No	289±116	6 weeks	increased hepatic transaminase concentration	No	0
[40]	Data from double-blind and open-label clozapine trials	USA	54 (34/20)	13.5±2.5 (range 7.0-19.1)	White:25, African American:17, Hispanic:4, Asian:2, Other:6	Childhood-onset, treatment resistant schizophrenia	Not mentioned	455.6±285.1 (n=46)	6 weeks	elevated liver enzymes (AST, ALP, ALT)(4%)	No	0
[53]	Cross-sectional	New Zealand	37 (29/8) [CLOZAPINE group: 20 (14/6) NON-CLOZAPINE group: 17 (15/2)]	39.3±9.8 (range 20-61) (CLOZAPINE group: 37±8.2, NON CLOZAPINE group:	Maori:12, Pacific islander:6, Pakeha (NZ European):2	Schizophrenia	Laxatives (laxsol, polyethylene glycol, lactulose), antipsychotics (risperidone:2, aripiprazole:1, haloperidol:1, amisulpride:7, aripiprazole+quetiapine:1) omeprazole, metformin, cholecalciferol *(*only 8 patients received clozapine as their sole antipsychotic)*	489±137 (range 284-885)	At least 3 months	Colonic Hypomotility	Positive correlation Clear colonic hypomotility in 80% of CLOZAPINE patients, with Colonic Transit Time (CCT) four times longer than population morms (p<0.0001) and NON CLOZAPINE patients (p<0.0001)	0
[54]	Retrospective, collection of records	UK	202 (141/61)	Males: 43.5±10.1 Females: 47.2±11.2	White: 82 Non-white: 120	Not reported	Additional anticholinergic agents (n=79)	Clozapineapine: Laxative users: 533±0.29 Non laxative users: 486±30 Norclozapine: Laxative users: 337±0.19, Non laxative users: 269±0.18	>3 months	Constipation as demonstrated by the use of laxatives	No correlation with clozapine plasma levels. Positive correlation with norclozapine	0

levels (laxative users had 29% higher levels of norclozapine compared to non users, p=0.046)

Table S7. Studies reporting hematological, genitourinary, and other adverse effects in relation to CLOZAPINE blood levels.

Reference	Type of study	Location	Patients (Males/ Females)	Age (Mean) (Years)	Race	Diagnosis	Comedication	Averaged serum levels of clozapine (ng/ml)	Duration	Reported side effects	Correlation to clozapine plasma levels	Jadad score
						Hematological System						
[41]	Cross-sectional	USA	44 (33/11)	35.6± 9.3 (range 17-54)	Not specified	Schizophrenia	Not reported	CLOZAPINE: 304±174 NCLOZAPINE:216±133	2.16± 0.35 years	WBC Neutrophil Count	No	0
[35]	Cross-sectional	Denmark	30 (21/9)	37,6 ± 1,67	Not specified	schizophrenia	Levomepromazine or chlorprothixene as sedatives in doses not exceeding 100 mg per day	Median 430.4 (282.4-752.8)	At least 3 months (range: 1-17 years)	Mild leukocytosis and increased erythrocyte folate	No	0
[37]	Prospective longitudinal	Canada	37 (26/11)	35.2±10.2 (range: 18- 57)	Not specified	Schizophrenia	Lorazepam 2 mg daily was used sparingly as needed for agitation and aggressive behaviour	379.5±156	4 to 8 weeks	Hematological parameters (WBC, red blood count, neutrophils, platelets, and lymphocytes counts or hemoglobin and hematocrit)	Weak, only with platelets count (small increase) between week 2 and 4 (p=0.042, r=0.32)	0
[40]	Prospective, double-blind (n=22), open-label (n=32)	USA	54 (34/20)	13.5±2.5 (range 7.0-19.1)	25 white, 17 African American, 4 Hispanic, 2 Asian 6 other	schizophrenia	Mood stabilizers and antidepressants	CLOZAPINE:455±285.1 NCLOZAPINE:302.4±142.2	6 week treatment (2-6 years follow-up)	Neutropenia (6%)	No	0
[43]	Prospective, Open-label/double blind	USA	6 (2/4)	13.3 ± 2.7 (range 9–16)	2 Caucasians, 2 African-Americans, 1 Hispanic and 1 Pacific Islander	Schizophrenia	None	CLOZAPINE:289±116 NCLOZAPINE: 410±190	6 weeks	Moderate neutropenia (one case)	No	0
[36]	Retrospective chart review	USA	58 (37/21)	36.4 ± 10.4	3 African-Americans, 55 white	Schizophrenia	Not reported	CLOZAPINE: 389±386 NCLOZAPINE:199±216	Mean duration not reported; Samples for	WBC or granulocyte counts	No	0

									measuring drug levels drawn At least once during the first 3 months and then randomly after			
[78]	Retrospective, observational	Norway	129 (77/52)	34 (median) 20-84 (range)	Not reported	Not reported	Not reported	CLOZAPINE: 1068 (26-3955) NCLOZAPINE: 712 (31-5813)	not reported	absolute neutrophil count (ANC)	Positive correlation between ANC and NCLOZAPINE levels (p=0.002) and NCLOZAPINE/CLOZAPINE ratio (p=0.04)	0
[77]	Cross-sectional, controlled	Turkey	70 Patients: 38 (17/21) Controls: (14/18)	Patients:40.94±10.15 Controls: 40.09±1.67	Not reported	Schizophrenia or schizoaffective	Not reported	594.90±492.90 220.33±182.55	At least 4 months	Hematological parameters	No correlation with RBC, MCV, MCH, MPV, WBC, LYM. Negative correlation with PLT (r=-0.362, p=0.025), HGB (r=-0.342, p=0.025), NEU (r=-0.385, p=0.017)	0
[38]	Prospective, open label	Italy	16 (12/4)	34.62±7.56 (range:25-48)	Not reported	schizophrenia	None	CLOZAPINE: 266.27±197.44 (25-1270) NCLOZAPINE: 169.0±127.94 (25-1280)	9 weeks	Leucocyte count Neutrophil count	Positive correlation between CLOZAPINE and NCLOZAPINE levels and neutrophil count (r=026, p=0.001, r=0.20, p=0.01, respectively). Negative correlation between NCLOZAPINE/CLOZAPINE ratio and neutrophil count (r=-0.26, p=0.002)	0
[69]	Prospective, open	Austria	68(42/26)	Males:28.9±9.7 Females:34.2±10.7	Not reported	Not reported	Not reported. Stated that patients with and without hematologic abnormalities did not differ in terms of comedications	145.8±160.1(3.1-1571.0)	16.7±24.7weeks	WBC disorders: Transient neutropenia (22%), eosinophilia (61.7%), and leukocytosis (40.9%). Progressive neutropenia (n=2)	No	0

									Chronic leukocytosis(n=1)		
[67]	Cross-sectional	Mexico	41 (22/19)	Males:34.68±1.58 Females: 31.58±1.29	Not reported	schizophrenia	Clonazepam: 30%, Fluoxetine: 22%, Paroxetine: 9%, Lithium: 7% Mirtazapine:6%, Metformin, Sulpiride, valproate, venlafaxine: 4% each, Duloxetine, escitalopram, imipramine, losartan, omeprazole, pregabalin:2% each	Males: 290.11±51.56 Females: 336.36±29.16	Males: 10.05±1.74 months Females: 6.63±1.15 months	Leukocyte count Neutrophil count	Negative correlation between CLOZAPINE levels and neutrophil (standardized coefficient:-0,631, p=0.004) and leukocyte count (standardized coefficient: -0.725, p=0.001). No correlation between NCLOZAPINE levels and neutrophil or leykocyte count	0
**Geniturinary Side Effects**											
[62]	Prospective, ,double-blind	USA	39 (gender Not reported)	Not reported	Not reported	Schizophrenia	Not reported	CLOZAPINE: 325±199 NCLOZAPINE:576±326 (dose-dependent)	16 weeks	Urinary disturbances (tested in association with antimuscrinic activity, highly correlated with CLOZAPINElevels, r=0.83, p<0.001)	No	2
[40]	Prospective, double-blind (n=22), open-label (n=32)	USA	54 (34/20)	13.5±2.5 (range 7.0-19.1)	25 white, 17 African American, 4 Hispanic, 2 Asian 6 other	schizophrenia	Mood stabilizers and antidepressants	CLOZAPINE:455±285.1 NCLOZAPINE:302.4±142.2	6 week treatment (2-6 years follow-up)	Enuresis (15%)	No	0
[43]	Prospective, Open-label/double blind	USA	6 (2/4)	13.3 ± 2.7 (range 9–16)	2 Caucasians, 2 African-Americans, 1 Hispanic and 1	Schizophrenia	None	CLOZAPINE:289±116 NCLOZAPINE: 410±190	6 weeks	Enuresis(1/6)	No	0

					Pacific Islander							
[41]	Cross-sectional	USA	44 (33/11)	35.6± 9.3 (range 17-54)	Not specified	Schizophrenia	Not reported	CLOZAPINE: 304±174 NCLOZAPINE:216±133	2.16± 0.35 years	Nocturnal Enuresis (27%)	No	0
[57]	Prospective	Austria	Sample on clozapine: 100 (75/25) (Total sample:153)	28.6±9.5	Not reported	Schizophrenia Schizophreniform disorder	Benzodiazepines, anticholinergic drugs, b-blockers, antidepressants, anticonvulsants	183.3±150.2	18 weeks	Sexual disturbances: Menorrhagia Amenorrhea Galactorrhea Dry Vagina Increased sexual desire Decreased sexual desire Orgasmic dysfunction Erectile dysfunction Ejaculatory dysfunction Gynecomastia	Positive in males, between clozapine plasma levels and diminished sexual desire (p=0.02) and functional disturbances (p=0.008)	0
						**Other**						
[55]	Clinical study	China	163 (47/116)	Group I: 38.8 (23-58), group II: 41.5 (21-58), group III: 43.9 (22-60)	Chinese	Schizophrenia	Not mentioned	221.4±109.6	38.3 ± 6.3 days (Clozapineapine group)	serum anticardiolipin antibodies (aCL) IgM, IgG	Positive correlation between CLOZAPINE levels and aCL IgM, (r=0.461, p=0.001) but no correlation with IgG	1
[77]	Cross-sectional, controlled	Turkey	70 Patients: 38 (17/21) Controls: (14/18)	Patients:40.94±10.15 Controls: 40.09±1.67	Not reported	Schizophrenia or schizoaffective	Not reported	CLOZAPINE:594.90±4NCLOZAPINE: 92.90 220.33±182.55	At least 4 months	B12, Na, K, ure, cre	No correlation with URE, CRE, Na, K, B12.	0

End of Supplemental data
